# Ecotoxicity evaluation of polymeric nanoparticles loaded with ascorbic acid for fish nutrition in aquaculture

**DOI:** 10.1186/s12951-021-00910-8

**Published:** 2021-05-31

**Authors:** Angélica I. S. Luis, Estefânia V. R. Campos, Jhones L. Oliveira, José Henrique Vallim, Patrícia L. F. Proença, Rodrigo F. Castanha, Vera L. S. S. de Castro, Leonardo F. Fraceto

**Affiliations:** 1 Institute of Science and Technology, Laboratory of Environmental Nanotechnology, São Paulo State University (UNESP), Sorocaba, SP 18087-180 Brazil; 2 Federal University of ABC, Santo André, SP 09210-580 Brazil; 3 Faculty of Agronomy and Veterinary Science, São Paulo State University (UNESP), Jaboticabal, SP 14884-900 Brazil; 4 Laboratory of Ecotoxicology and Biosafety, Embrapa Environment, Jaguariúna, SP 13918-110 Brazil

**Keywords:** Chitosan nanoparticles, Polycaprolactone nanoparticles, Vitamin, Zebrafish, Ecotoxicity

## Abstract

**Background:**

Ascorbic acid (AA) is a micronutrient essential for the mechanisms of reproduction, growth, and defense in fish. However, the biosynthesis of this micronutrient does not occur in fish, so it must be supplied with food. A difficulty is that plain AA is unstable, due to the effects of light, high temperature, and oxygen, among others. The use of nanoencapsulation may provide protection and preserve the physicochemical characteristics of AA for extended periods of time, decreasing losses due to environmental factors.

**Method:**

This study evaluated the protective effect of nanoencapsulation in polymeric nanoparticles (chitosan and polycaprolactone) against AA degradation. Evaluation was made of the physicochemical stability of the nanoformulations over time, as well as the toxicological effects in zebrafish (*Danio rerio*), considering behavior, development, and enzymatic activity. For the statistical tests, ANOVA (two-way, significance of p < 0.05) was used.

**Results:**

Both nanoparticle formulations showed high encapsulation efficiency and good physicochemical stability during 90 days. Chitosan (CS) and polycaprolactone (PCL) nanoparticles loaded with AA had mean diameters of 314 and 303 nm and polydispersity indexes of 0.36 and 0.28, respectively. Both nanosystems provided protection against degradation of AA exposed to an oxidizing agent, compared to plain AA. Total degradation of AA was observed after 7, 20, and 480 min for plain AA, the CS nanoparticle formulation, and the PCL nanoparticle formulation, respectively. For zebrafish larvae, the LC_50_ values were 330.7, 57.4, and 179.6 mg/L for plain AA, the CS nanoparticle formulation, and the PCL nanoparticle formulation, respectively. In toxicity assays using AA at a concentration of 50 mg/L, both types of nanoparticles loaded with AA showed lower toxicity towards the development of the zebrafish, compared to plain AA at the same concentration. Although decreased activity of the enzyme acetylcholinesterase (AChE) did not affect the swimming behavior of zebrafish larvae in the groups evaluated, it may have been associated with the observed morphometric changes, such as curvature of the tail.

**Conclusions:**

This study showed that the use of nanosystems is promising for fish nutritional supplementation in aquaculture. In particular, PCL nanoparticles loaded with AA seemed to be most promising, due to higher protection against AA degradation, as well as lower toxicity to zebrafish, compared to the chitosan nanoparticles. The use of nanotechnology opens new perspectives for aquaculture, enabling the reduction of feed nutrient losses, leading to faster fish growth and improved sustainability of this activity.

**Graphic Abstract:**

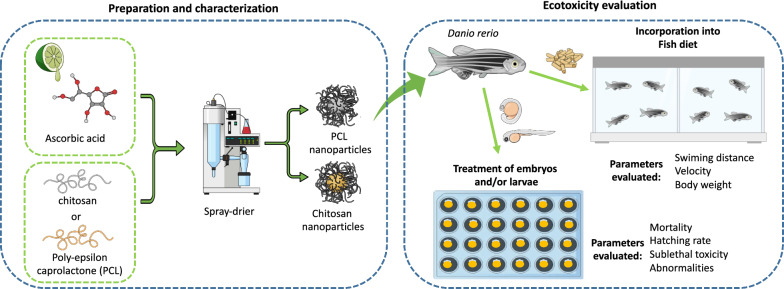

**Supplementary Information:**

The online version contains supplementary material available at 10.1186/s12951-021-00910-8.

## Introduction

With the global population predicted to increase by approximately 30% to 9.7 billion in 2050, one of the greatest challenges currently facing the world is food security [1]. Aquaculture can actively contribute to ensuring an adequate food supply, providing high quality protein to feed the growing world population, especially in developing countries [2]. However, in efforts to maximize production, the aquaculture sector faces challenges regarding the emergence of diseases, which are very common in intensive production systems [3]. Therefore, it is necessary to develop suitable strategies that can ensure the sustainable development of aquaculture [4]. The Blue Growth initiative aims at the sustainable global development of aquaculture, enabling the sector to contribute to the Sustainable Development Goals (SDGs) of the United Nations, especially Goals 2 and 14. Goal 2 concerns the achievement of zero hunger, food security, and improved nutrition, while Goal 14 aims at the conservation and sustainable development of the oceans, seas, and marine resources [5, 6].

Among the strategies for the development of sustainable aquaculture, the utilization of natural sources of vitamins to improve the growth and development of aquatic species has been studied [2, 7, 8]. Vitamins act mainly as cofactors of enzymes, so their inadequate supplementation can be detrimental to organism growth and survival, with increased vulnerability to diseases and infections [9, 10]. Due to their coenzyme activities, vitamins contribute to normal metabolic functioning and the maintenance of health [8]. The hydrophilic vitamin L-ascorbic acid (AA) is an essential micronutrient required by aquatic animals, since they do not have the ability to synthesize it [7, 8].

It has been reported that dietary supplementation with AA can positively affect growth performance, immunological responses, and survival rate, as well as increase the resistance to stressors in different fish species [8]. The amount of AA required in the diet varies according to species, size, age, breeding condition, and the form of AA administered [3]. In its pure form, ascorbic acid is unstable in alkaline environments, where its oxidation is accelerated, leading to its loss at rates influenced by factors including temperature, oxygen, light, and pH, among others [11]. Therefore, it is important to develop new technologies capable of protecting this molecule against premature degradation, increasing its stability and bioavailability.

Nanoencapsulation offers a way to improve the stability of AA [12, 13]. The materials used for nanoencapsulation include a variety of matrices that may have natural or synthetic origins [14]. One such material, chitosan, is derived from chitin, obtained mainly from crustaceans such as crabs, although this polymer is also found in the cell walls of fungi. After being separated, chitin is submitted to an alkalinization or acidification process. Chitosan easily penetrates cell and transcellular walls, is positively charged, forms complexes by hydrophobic interactions or hydrogen bonds, and is soluble in acidic media [15]. In fish, chitosan can adhere to the gills and skin, due to the interaction between chitosan nanoparticles and the layer of mucopolysaccharides and mucoproteins that covers the organism [16]. Other polymer widely used to nanoencapsulation is poly (ε-caprolactone), which is an aliphatic polyester, biodegradable and biocompatible. It is a FDA (US Food and Drug Administration) approved polymer with high permeability to many compounds, non-toxicity and reasonably low cost that renders this polymer attractiveness for aquaculture applications [17].

Despite the potential benefits of nanocarrier systems, concerns have been raised about the biosafety of their use in aquaculture. Therefore, it is necessary to evaluate and characterize the possible ecotoxicological risks resulting from their use, in order to make decisions about the implementation of these nanotechnologies in agricultural practices [18]. Hence, it is essential to understand the potential effects of nanocarrier systems towards both target and nontarget organisms. For this purpose, the zebrafish (*Danio rerio*) has been used to assess the effects of different compounds on the fish community [19]. Monitoring of the development of *Danio rerio* provides a rapid way to evaluate the environmental risks of nanomaterials [20].

Decrease the premature degradation of AA, the aim of this work was to prepare and characterize polymeric nanoparticles (chitosan and polycaprolactone) used to encapsulate AA, followed by evaluation of their effects on the development of zebrafish embryos and larvae. In addition, nanoparticulate systems with or without AA were evaluated for possible toxic effects on the development of these organisms. Different parameters of physical development were evaluated, including malformations, hatching and survival rates, and behavioral indicators. The AchE activity was also determined, since its alteration can lead to impaired motor function [21]. This study opens perspectives for the development of systems for fish nutrition in aquaculture, aiming at more sustainable production that ensures the quality of life of the organisms.

## Materials and methods

### Materials

L-ascorbic acid (99%), trehalose dihydrate, poly-ε-caprolactone, and chitosan (50,000–190,000 Da) were obtained from Sigma-Aldrich and were used without further purification. Acetonitrile and methanol (HPLC grade) were obtained from J.T. Baker. Low molecular weight chitosan and the chemicals used in the oxidation assays (Oxone**®** and EDTA) were obtained from Sigma-Aldrich. The AchE assays employed acetylthiocholine iodide and DTNB (5,5′-dithiobis (2-nitrobenzoic acid) acquired from Sigma-Aldrich.

### Nanoparticles synthesis

#### Chitosan nanoparticles

Nanoparticles composed of chitosan and sodium tripolyphosphate (TPP) were prepared by the ionic gelation technique at room temperature (25ºC), followed by spray drying, according to [22]. A 250 mL volume of an aqueous solution of acetic acid (0.5% v/v) was prepared containing low molecular weight chitosan (1% w/v). Separately, 10 mL of an aqueous solution was prepared containing 2.5 g of AA. The AA solution was added to the chitosan solution and the resulting mixture was homogenized for 10 min, under magnetic stirring at 800 rpm. This solution was then dripped into 10 mL of TPP solution (1% w/v) under magnetic stirring at 800 rpm for 10 min. The final concentration of AA was 0.01 mg/mL. Control nanoparticles were prepared using the same method described above, without addition of AA. In order to guarantee the nanoparticles dispersibility and their regular spherical shape, trehalose dihydrate (1% w/v) was used as additional excipient [23] The nanoparticles were dried using a laboratory spray dryer with a 1 mm diameter nozzle (Model MSD 1.0, Labmaq, Brazil). The operating conditions were inlet temperature at 110.4 °C, outlet temperature at 72 °C, feed rate of 0.3 L h^−1^, atomization flow of 40 L h^−1^, and drying flow of 1.50 m^3^ min^−1^. The powder obtained was stored at room temperature until use.

#### Polycaprolactone nanoparticles

Polycaprolactone nanoparticles were prepared by the double emulsion-evaporation method at room temperature (25ºC), with slight modifications [24]. For the organic phase, 400 mg of PCL were dissolved in 20 mL of dichloromethane. The aqueous phase was PVA (3 mg mL^−1^) dissolved in ultrapure water. AA was dissolved in ethanol and mixed with the aqueous phase containing PVA, followed by ultraturrax homogenization for 1 min at 14,000 rpm. The resulting mixture was added to the organic phase and homogenized for 8 min by ultraturrax at 14,000 rpm. The organic solvent was evaporated using a rotary evaporator and the formulation was concentrated to a volume of 10 mL. The final concentration of AA was 5 mg mL^−1^. The nanoparticles were dried using a laboratory spray dryer with a 1 mm diameter nozzle (MSD 1.0, Labmaq, Brazil). In order to guarantee the nanoparticles re-dispersibility and their regular spherical shape, trehalose dihydrate (1%) was used as additional excipient [23]. The operating conditions were inlet temperature at 110.4 °C, outlet temperature at 65.4 °C, feed rate of 0.3 L h^−1^, atomization flow of 40 L h^−1^, and drying flow of 1.50 m^3^ min^−1^. The powder obtained was stored at room temperature until use.

### Nanoparticles characterization

Evaluation was made of the physicochemical characteristics of both nanoparticle formulations, including the mean diameter, polydispersity index, zeta potential, pH, and encapsulation efficiency. For these analyses, the dried nanoparticles (10 mg) were resuspended in 1 mL of ultrapure water. The samples were stored in amber flasks at room temperature, protected from light.

### Mean diameter and polydispersity index

The mean diameter and polydispersity index were determined by the photon correlation spectroscopy technique, using a ZetaSizer Nano ZS 90 instrument (Malvern Instruments) at an angle of 90°. The nanoparticle solutions were diluted 1,500 times with deionized water. The measurements were performed in triplicate (n = 3).

### Zeta potential and pH

The surface charges of the nanoparticles were determined by zeta potential measurements using the microelectrophoresis technique, employing a ZetaSizer Nano ZS 90 analyzer (Malvern Instruments). The samples were diluted 1,500 times and analyzed in triplicate. The pH values of the different formulations were obtained using a potentiometer (Tecnal) previously calibrated with pH 7 and pH 4 standards.

### Ascorbic acid (AA) encapsulation efficiency

The AA encapsulation efficiency was determined by the ultrafiltration-centrifugation method. The nanoparticles were centrifuged using a 10 kDa cellulose filter (Millipore) that allowed the passage of non-encapsulated AA. The encapsulation efficiency was determined as the difference between the amounts of AA added to the formulation (100%) and present in the filtrate (non-encapsulated AA). The quantification of AA was performed by HPLC, with UV detection at 250 nm. The separation was performed using a HyperClone C18 column (250 × 4.6 mm, 5 μm), with a mobile phase consisting of a 95:5 (v/v) mixture of methanol and 1% (v/v) acetic acid. Quantification employed an analytical curve (y = 1.99x – 5.83) with correlation coefficient of 0.99903, limit of detection (0.277 µg mL^−1^) and quantification (0.926 μg mL^−1^).

### AA release kinetics and mathematical modeling

The profiles of AA release from the nanoparticles were evaluated using a two-compartment system composed of a donor compartment (2 mL) and an acceptor compartment (50 mL), separated by a cellulose membrane (1000 Da exclusion pore size) under slight stirring and room temperature (25 ºC). Aliquots (1 mL) were periodically collected from the acceptor compartment, with the volume being replaced with 1 mL of water to maintain a constant volume. The aliquots removed were analyzed by HPLC and the results were converted to percentages of released AA. The assays were performed in triplicate. Zero order, first order, Higuchi and Korsmeyer-Peppas mathematical models were used to elucidate the release mechanism of AA from all formulations [25].

### AA oxidation kinetics

The oxidation profiles of AA, alone and encapsulated in the CS and PCL nanoparticles, were evaluated using the method described previously [26]. A stock solution of AA was prepared by dissolving 50 mg of AA in 250 mL of stabilizing solution (EDTA). The absorbance of this solution was measured at 265 nm, with the stabilizing solution used as the background. Separately, a solution of Oxone (potassium peroxymonosulfate, Sigma-Aldrich) was prepared in 50 mL of EDTA solution. An aliquot of AA solution was mixed with 3 mL of Oxone solution and completed to 25 mL with the stabilizing solution, resulting in a solution with 10 μg mL^−1^ of AA. The same procedure was performed for the CS and PCL nanoparticles loaded with AA. The absorbance value of the solution containing only AA, minus the absorbance value of the solution containing AA and Oxone, was proportional to the concentration of AA present in the sample. For oxidation studies under aquarium conditions, 9 aquaria were used (3 per treatment), each containing a final volume of 3 L and fitted with an aeration pump. The parameters set for the experiment were 28 °C, pH 7 ± 0.5, and 25 µg mL^−1^ of AA (the concentration used in the in vivo assays). At predetermined times, 1 mL aliquots were collected, and the amount of AA was determined using HPLC. The results were presented as % viable ascorbic acid. The assays were performed in triplicate (n = 3).

### In vivo* studies*

#### Maintenance and reproduction of the animals

The breeding fish (*Danio rerio*, wild-type strain) were kept in a Rack Hydrus system (Model ZEB-40, Alesco), under controlled conditions of 28 ± 0.2 ºC, conductivity of 400 ± 0.2 µS, and pH 7.0 ± 0.2. The pH was controlled using solutions of 30 g L^−1^ sodium bicarbonate (Synth) and 1 mL L^−1^ HCl (37%, ACS, Scharlau). The conductivity was adjusted with 30 g/L saline solution (Red Sea salt or Instant Ocean). The embryos were kept in reconstituted water prepared according to the protocol of the USEPA (2002). The experiments carried out in this work were approved by the Animal Use Ethics Committee (CEUA) of Embrapa Environment (protocol nº 12/2018).

#### Toxicity evaluation using zebrafish

The embryos and larvae were divided into control groups (embryo medium only) and treatment groups with addition of ascorbic acid (AA), empty chitosan nanoparticles (NPs_CS), chitosan nanoparticles loaded with AA (NPs_CS_AA), PCL nanoparticles (NPs_PCL), and PCL nanoparticles loaded with AA (NPs_PCL_AA). Preliminary tests were performed to determine the lethal concentration (LC_50_, mg/L). For this experiment, 24-well polystyrene plates were used, with the embryos being individually exposed to 2 mL of the test solution (n = 24 per group). The concentrations used for each exposure were 3.12, 6.25, 12.5, 25, 50, 100, 200, and 400 mg/L. The same dilutions were used for the control nanoparticles without the presence of AA. The positive control was 4 mg L^−1^ of 3,5-dichloroaniline (≥ 98%, Sigma-Aldrich). The plates were examined every 24 h to determine the mortality of the embryos and larvae, using a stereomicroscope (Model SMZ 2 LED, Optika) and Optika View v. 7.1.1.5 software. After 96 h of exposure, the LC_50_ was determined for each group, using probit analysis performed with Statgraphics Centurion XVII v. 1.17.04 software.

#### Evaluation of fish embryo acute toxicity (FET)

The embryos were exposed to different concentrations (LC_10_, LC_20_, LC_30_, LC_40_, and LC_50_) of the treatments (n = 24 per group). The exposure was carried out for 96 h under the test conditions described in OECD protocol 236 (OECD, 2013). The embryos were kept individually in 24-well polystyrene plates (n = 24 organisms per group), with addition of 2 mL of test solution to each well. The temperature was maintained at 26 ± 0.2 °C and a light/dark photoperiod of 14/10 h was provided. Determinations were made of mortality and morphological abnormalities including changes in heartbeat, presence of coagulation, undetected tail, change in head formation, disintegration of larvae, pericardial edema, yolk sac edema and opacity, and defective development of the somite.

The total length of the larvae (mm) was measured from the mouth to the terminal portion of the tail. The embryos and larvae were evaluated every 24 h, using a stereomicroscope to determine the occurrence of malformations and/or mortality. After exposure for 96 h, the live larvae were photographed and their total lengths were measured using Optika View v. 7.1.1.5 software previously calibrated with a millimeter scale.

Mortality was defined as the presence of coagulation, lack of heartbeat, failure of somite development, and undeveloped tail. The hatching rate was calculated considering the successful hatching of embryos in relation to the total number of embryos in each replicate. The observed malformations included pericardial edema, tail deformity, yolk sac edema, and spinal curvature. Pericardial edema was identified as swelling due to an increase in the volume of fluid in the pericardium, which is the portion of the coelomic cavity that separates the heart from the body wall [27]. The presence or absence of an inflated swimming bladder was determined for the embryos 120 h post-fertilization (hpf), using a microscope.

The malformation rate was determined considering the percentage of malformed larvae in relation to the total number of larvae that hatched during the test. At the end of the exposures (96 h), the larvae (n = 10 per group) were photographed and the total length was measured using the Optika View v. 7.1.1.5 software, at 2 × magnification. The equipment was previously calibrated using a millimeter scale.

#### Evaluation of zebrafish motor activity

For evaluation of the behavioral alterations caused by the different treatments, the embryos were divided into the following groups: control (embryo medium only), ascorbic acid (AA), chitosan nanoparticles (NPs_CS), chitosan nanoparticles loaded with AA (NPs_CS_AA), PCL nanoparticles (NPs_PCL), and PCL nanoparticles loaded with AA (NPs_PCL_AA). Zebrafish embryos (n = 36 per group) were exposed (96 h) under sublethal and acute toxicity conditions, using the different treatments at concentrations of LC_10_, LC_20_, LC_30_, LC_40_, and LC_50_. After 96 h, the larvae were distributed individually in 96-well plates, with each well containing 100 µL of embryo medium and 100 µL of test solution (total volume = 200 µL). The behaviors of the larvae were recorded individually for 10 min (at 28 °C) and were analyzed using EthovisionXT software (Noldus). The parameters considered were speed (mm/s) and distance covered.

#### Evaluation of acetylcholinesterase (AChE) activity

AChE activity was determined according to the method described previously [28]. The embryos (2 hpf) were exposed for 96 h to different concentrations (LC_10_, LC_20_, LC_30_, LC_40_, and LC_50_) of the treatments: control (embryo medium only), ascorbic acid (AA), chitosan nanoparticles (NPs_CS), chitosan nanoparticles loaded with AA (NPs_CS_AA), PCL nanoparticles (NPs_PCL), and PCL nanoparticles loaded with AA (NPs_PCL_AA). The exposures were performed in sterile polystyrene Petri dishes. Live larvae were collected 96 h after the start of exposure, for the determination of AChE activity. The larvae were washed (3 times) with cold (4 °C) phosphate buffer (0.5 mol/L) at pH 7.0, followed by storage in Eppendorf microtubes containing 0.5 mL of phosphate buffer (n = 5 pools of 10 larvae per group). The readings were performed in triplicate. The samples in phosphate buffer were homogenized with an ultraturrax, followed by centrifugation for 10 min at 10,000 g and 4 °C. The supernatant was used for the analysis of AChE. The protein concentrations in the samples were determined as described previously by Bradford [29]. Five pools of 10 larvae were analyzed for each group, with the readings performed in triplicate. A 96-well microplate spectrophotometer (Sunrise, Tecan) was used for the analysis. The AChE activity was determined as described previously by Elman et al*.,* [28], modified for microplates [30]. For this, 15 μg of the homogenate was added to 15 µL of 9 mmol/L acetylcholine (acetylthiocholine iodide, Sigma-Aldrich) and 70 µL of 0.75 mmol/L DTNB (5.5′-dithiobis (2-nitrobenzoic acid), Sigma-Aldrich). The absorbance at 412 nm was monitored for 5 min, at 30 °C (11 cycles, with 30 s between readings).

#### Evaluation of zebrafish body weight

Seventy zebrafish larvae with initial weight 1.8–1.9 mg and length 7.748–8.398 mm were distributed in 7 aquaria with volumes of 3 L (n = 10 organisms per group) and were exposed to the different treatments for 61 days. The control group was fed with commercial food (TetraMIN), while the other groups were fed with TetraMIN plus different concentrations (25, 50, and 100 mg/kg) of chitosan nanoparticles loaded with AA (NPs_CS_AA) or PCL nanoparticles loaded with AA (NPs_PCL_AA). The fish were fed to satiety, three times a day. At the end of the experimental period, the fish were fasted for 24 h. After this period, individual measurements were made of the weights and total lengths of the fish from each treatment. The results were presented as the means of the final weights and lengths of 10 organisms.

## Results and discussion

### Characterization of the polymeric nanoparticles

Chitosan nanoparticles cross-linked with sodium tripolyphosphate were prepared by the ionic gelation method, while polycaprolactone nanoparticles were synthesized using the emulsification/evaporation method. In both cases, the synthesis was followed by spray drying. The physicochemical properties of the nanoparticles were determined before and after the spray drying process.

For the chitosan nanoparticles (NPs_CS) and the chitosan nanoparticles containing AA (NPs_CS_AA) in aqueous suspension, the average diameters were 316 ± 5 nm and 337 ± 3 nm, respectively. The polydispersity indexes for these nanoparticles ranged from 0.39 to 0.40, while the zeta potentials were between 47 and 76 mV (Table 1).

**Table 1:** Physicochemical characterization of the nanoparticles loaded with AA

Samples	MD (nm)	PDI	ZP (mV)	EE (%)
Nanoparticles before spray drying				
NPs_CS	316 ± 5	0.39 ± 0.13	47 ± 0.7	–
NPs_CS_AA	337 ± 3	0.40 ± 0.01	76 ± 0.5	98 ± 0.1
NPs_PCL	329 ± 1	0.23 ± 0.10	− 19 ± 0.2	–
NPs_PCL_AA	350 ± 5	0.34 ± 0.10	− 20 ± 0.1	95 ± 0.4
Nanoparticles after spray drying				
NPs_CS_SD	294 ± 1	0.33 ± 0.02	41 ± 0.5	–
NPs_CS_AA_SD	314 ± 3	0.36 ± 0.01	60 ± 1.7	96 ± 0.5
NPs_PCL_SD	281 ± 1	0.21 ± 0.05	− 15 ± 0.2	–
NPs_PCL_AA_SD	303 ± 1	0.28 ± 0.06	− 18 ± 0.4	91 ± 0.2

The parameters determined were the mean particle diameter (MD) obtained by the dynamic light scattering method, polydispersity index (PDI), zeta potential (ZP), and encapsulation efficiency (EE). The values are the means of three determinations

For the PCL nanoparticles (NPs_PCL) and the PCL nanoparticles containing AA (NPs_PCL_AA) in aqueous suspension, the average diameters were 329 ± 1 nm and 350 ± 5 nm, respectively. The polydispersity indexes were between 0.23 and 0.34, while the zeta potentials for these nanoparticles were negative, with values of -19 ± 0.2 mV for NPs_PCL and  − 20 ± 0.1 mV for NPs_PCL_AA (Table 1). Both nanoparticulate systems showed high encapsulation efficiencies, with values of 98 ± 0.1% for NPs_CS_AA and 95 ± 0.4% for NPs_PCL_AA. However, there were significant decreases in the encapsulation efficiency during storage (data not shown), which could be attributed to the high aqueous solubility of AA (330 g/L). Furthermore, it is known that AA presents rapid degradation in aqueous solution. For these reasons, together with the fact that the objective of this work was to prepare nanoparticles to be incorporated into fish diets, it was decided to submit these nanoparticles to a spray drying process, in order to extend the stability of the AA and improve its biological activity.

**Fig. 1:**
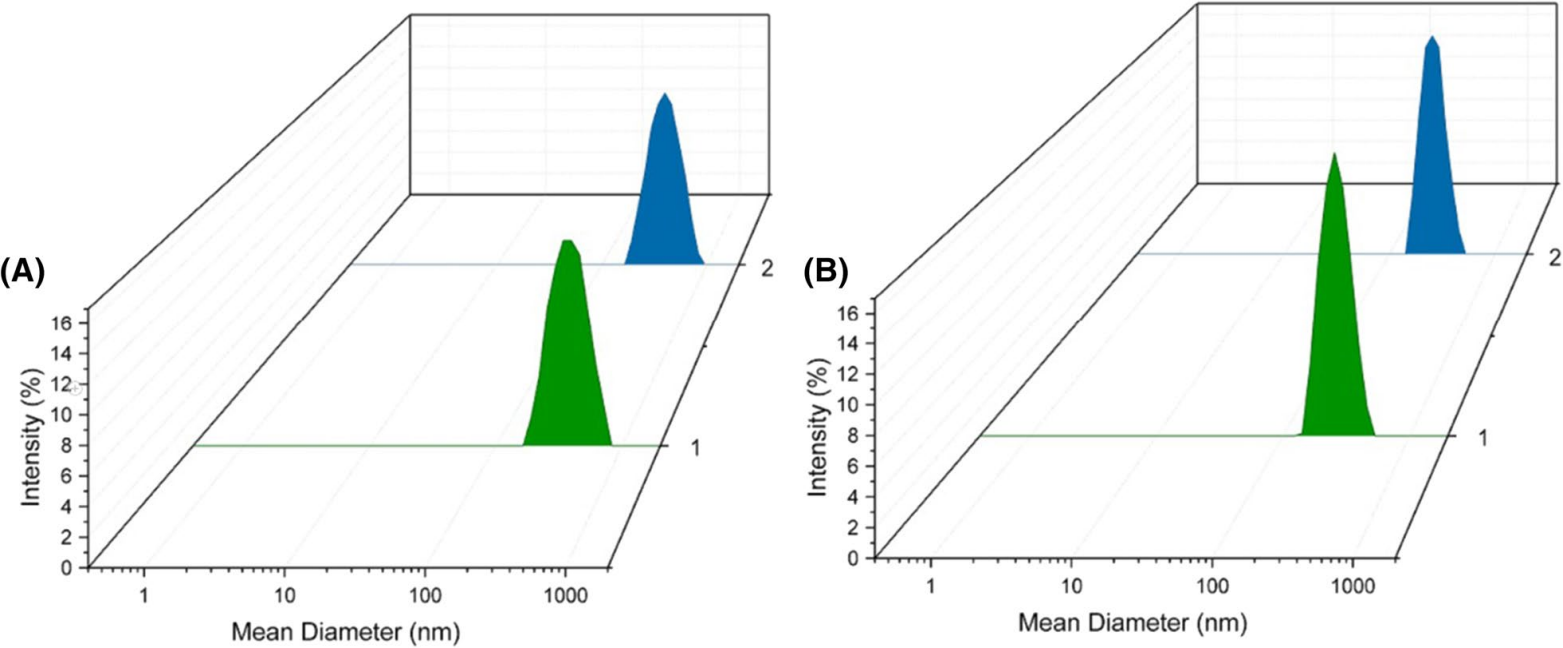
Size distributions obtained using the DLS technique. **A** Chitosan nanoparticles loaded with AA, before the spray drying process (NPS_CS_AA) (1) and after spray drying (NPs_CS_AA_SD) (2). **B** PCL nanoparticles containing AA, before spray drying (NPs_PCL_AA) (1) and after spray drying (NPs_PCL_AA_SD) (2)

After drying the nanoparticles, they were resuspended in distilled water and their physicochemical characteristics were determined, in order to evaluate the effect of the drying process. The average diameters of the chitosan nanoparticles (NPs_CS_SD) and the chitosan nanoparticles containing AA (NPs_CS_AA_SD) presented reductions of 22 and 23 nm, respectively. Decreases of the polydispersity index were also observed for both formulations, although the chitosan nanoparticles loaded with AA showed the same size distribution pattern before and after the spray drying process (Fig. 1A). Chitosan nanoparticles are electrostatically stabilized, so the strong cationic charge of the nanoparticles produced could be an indication of good stability of the nanoformulation. Another important parameter that did not undergo significant alteration was the efficiency of encapsulation of AA in the chitosan nanoparticles, with a value of 96 ± 0.5% obtained.

Similar to the behavior observed for the chitosan nanoparticles, the dried PCL nanoparticles showed reductions of 48 and 47 nm for NPs_PCL_SD and NPs_PCL_AA_SD, respectively (Table 1). Decreases of the polydispersity index also occurred after the drying processes, but the size distribution was not modified, as shown in Fig. 1B. The zeta potentials showed small increases, compared to the nanoparticles before the drying process, but remained negative, which is a characteristic of PCL. The zeta potential values for these nanoparticles were below the values recommended in the literature for a stable colloidal system. It is well known that the zeta potential is responsible for the colloidal stabilization of the nanoparticles in suspension, preventing their aggregation, due to the electrostatic repulsion of the particles, thus, the higher the value of the zeta potential (in module), the greater the electrostatic repulsion and the lower the tendency to form aggregates. However, in the case of the PCL formulations, PVA was used as a stabilizing agent, with the main stabilization mechanism of this surfactant involving steric hindrance, rather than electrostatic charge effects.

A high AA encapsulation efficiency (91 ± 0.2%) was observed, indicative of strong affinity of the compound for the nanoparticles. The physicochemical characterization data demonstrated that the drying process did not cause changes capable of destabilizing the system, indicating that this process could be applied to both types of nanoparticle, in order to improve the stability of AA. All the results presented below were performed using nanoparticles that were spray dried and subsequently resuspended before each experiment.

### Kinetics of AA release from the nanoparticles

**Fig. 2:**
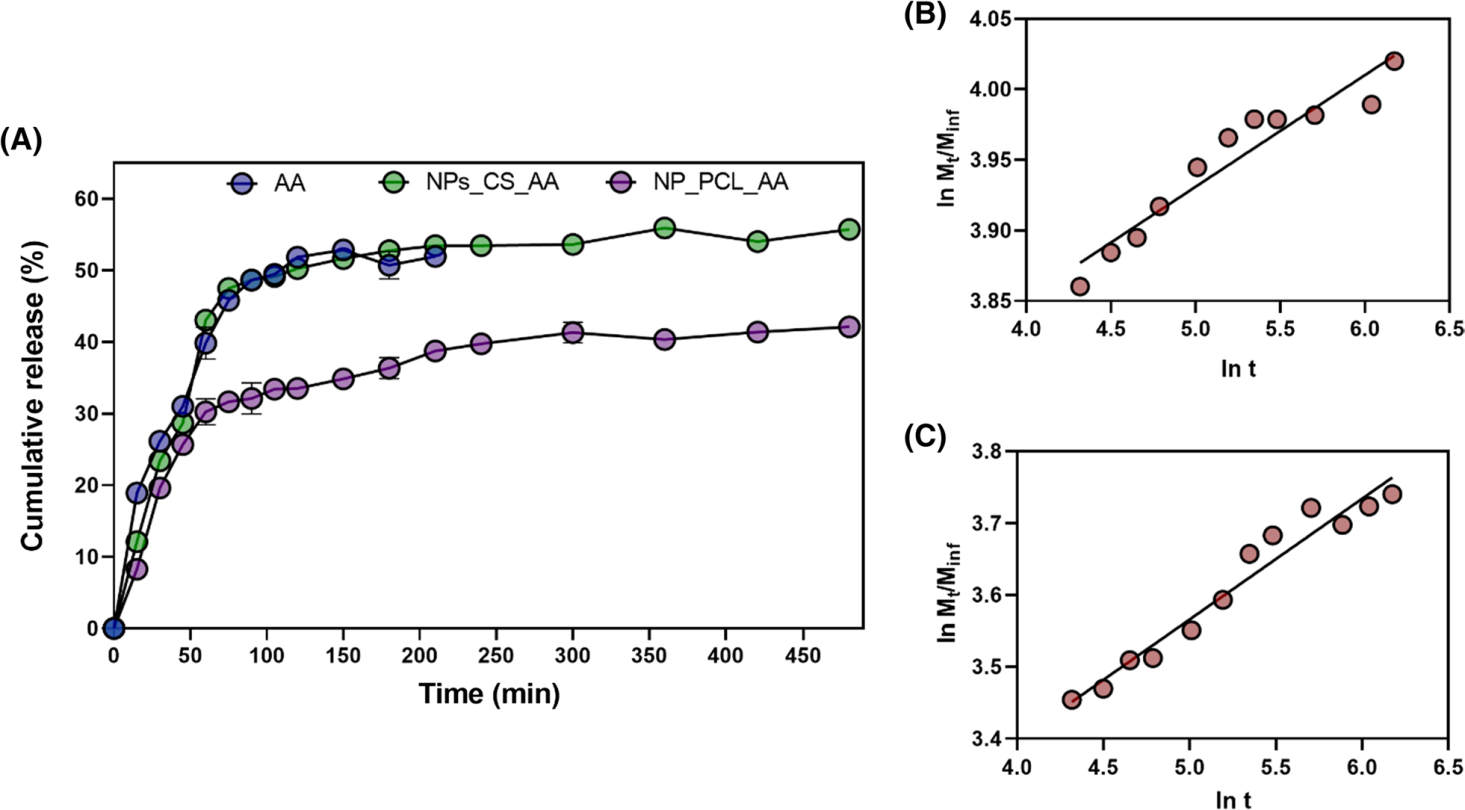
**A** In vitro release kinetics profiles for ascorbic acid (AA), chitosan nanoparticles containing ascorbic acid (NPs_CS_AA), and PCL nanoparticles containing AA (NPs_PCL_AA), for analyses performed in triplicate, with quantification by HPLC. Fitting of the Korsmeyer-Peppas mathematical model for the release of AA from **B** NPs_CS_AA and **C** NPs_PCL_AA

The profiles of release of AA from the chitosan nanoparticles (NPs_CS_AA) and the PCL nanoparticles (NPs_PCL_AA) are shown in Fig. 2A. For non-encapsulated ascorbic acid, the concentration of this compound in the acceptor compartment was 39 ± 0.2% after 60 min, reaching 51 ± 0.9% after 210 min, followed by a decrease of the concentration, due to degradation of the compound in the aqueous medium, which precluded further concentration measurements for this system.

The release patterns observed for NPs_CS_AA and NPs_PCL_AA were similar, with faster release of AA in the first hour of the experiment, followed by a more constant release (Fig. 2A). After 60 min, releases of 43 ± 0.1% and 30 ± 0.1% were observed for NPs_CS_AA and NPs_PCL_AA, respectively. In comparison with the non-encapsulated AA, the chitosan nanoparticles were not able to modify the AA release profile, with maximum release of 55.7 ± 0.03% after 480 min. In contrast, the association of AA with the PCL nanoparticles significantly delayed the release of AA, with release of 42 ± 0.03% of the encapsulated AA after 480 min. A previous study evaluated the release of AA from chitosan nanoparticles in seawater (pH 8), with only 6.57 ± 0.52% of the AA released after 120 min, which was 7.6 and 5 times less than observed here for the chitosan and PCL nanoparticles, during the same period of time [31]. The lower release of AA observed by these authors can be explained by the difference of the nanoparticulate system. In this system, there is the presence of cyclodextrin, thus, AA is complexed with cyclodextrin, which was further involved by CS. In this way, the AA needs to diffuse from the CD and later, from the CS to be released to the medium,thus, the 6.57 ± 0.52% release refers to the AA that is mainly dispersed close to nanoparticles surface.

The mechanisms of release of AA from the chitosan and PCL nanoparticles were investigated by application of the zero order, Higuchi, and Korsmeyer-Peppas mathematical models. The Korsmeyer-Peppas model provided the best fit to the release data for both the CS and PCL nanoparticles, with r^2^ values of 0.9189 and 0.9433 for NPs_CS_AA and NPs_PCL_AA, respectively (Fig. 2B, C). For both systems, the release exponent (n) value was less than 0.5 (n = 0.28 for the chitosan nanoparticles and n = 0.38 for the PCL nanoparticles), indicating that the release mainly occurred by diffusion of AA through the swollen polymeric matrix and through the hydrophilic pores (Fickian diffusion). The release constants (k) for the chitosan and PCL nanoparticles were 0.1899 and 0.07912 min^−1^, respectively, indicating that faster release of AA occurred from the CS nanoparticles, compared to release from the PCL nanoparticles. Brito & colleagues evaluated the release of hydro soluble vitamins from chitosan nanoparticles. The release constant (k) for vitamin C released from chitosan nanoparticles at pH 7.4 and pH 3 were 1.0 and 1.4 min^−1^ respectively [32]. In other hand, when the release rate of vitamin C was evaluated from polycaprolactone-polyethylene glycol microparticles in three different pH (2.8, 7.4 and 9.6), the obtained values were between 0.0127 to 0.0493 min^−1^ [33]. In this way, the results found here corroborate with previous published results in the literature, where the release rate of AA is faster when encapsulated into chitosan nanoparticles in comparison to PCL nanoparticles.

### AA oxidation kinetics

**Fig. 3:**
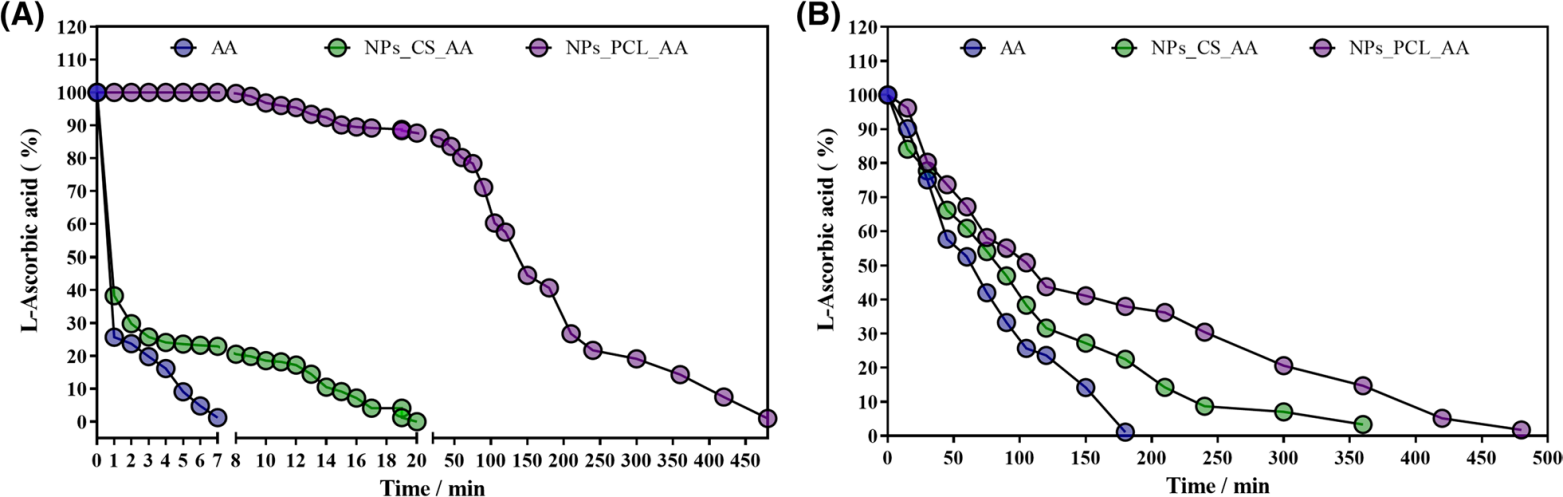
Oxidation of non-encapsulated ascorbic acid (AA) and ascorbic acid encapsulated in chitosan nanoparticles (NPs_CS_AA) and PCL nanoparticles (NPs_PCL_AA). The oxidation of ascorbic acid was **A** induced using peroxymonosulfate and **B** occurred under aquarium conditions simulating the natural environment of fish

The oxidation of AA can occur according to both anaerobic and aerobic mechanisms, depending on the availability of oxygen. In the non-oxidative pathway, AA is hydrolyzed to produce an acyclic structure. In the oxidative pathway, oxidation occurs due to the presence of oxygen, producing dehydroascorbic acid [34]. The oxidation of ascorbic acid was induced by the presence of peroxymonosulfate, resulting in a two-stage reaction in which an ascorbate free radical is firstly produced, followed by the donation of a second electron to produce dehydroascorbic acid, in a reversible process. Dehydroascorbic acid undergoes a ring opening reaction, resulting in 2,3-diketogulonic acid and causing loss of vitamin activity [26]. Figure 3 shows the oxidation profiles of non-encapsulated AA and AA encapsulated in the chitosan or PCL nanoparticles.

For the non-encapsulated AA, addition of the oxidizing agent to the solution resulted in a fourfold decrease (2.5 ± 0.01 µg/mL) of the AA concentration, relative to the initial value, in the first minute of the experiment (Fig. 3A). Total degradation of the AA present in the solution was observed after 7 min. The AA present in the chitosan NPs also showed rapid (2.6-fold) degradation during the first minute of the experiment, in the presence of the oxidizing agent. Nonetheless, the encapsulation of AA in the chitosan NPs was able to decrease the degradation rate, which stabilized between 2 and 8 min, followed by progressive degradation until complete removal of the AA after 20 min. Encapsulation of AA in the PCL NPs resulted in much slower degradation of the compound, with total degradation only reached after 8 h.

In addition, the degradation of AA, free or encapsulated in the CS and PCL nanoparticles, was evaluated in aquarium simulations representing the normal conditions of the fish environment (Fig. 3B). This experiment with a constant aeration system simulated the natural degradation of AA in aquaculture systems, where there is continuous movement of the water. It has been reported that greater movement of water (solvent) accelerates the degradation of AA [35].

As shown in Fig. 3B, faster degradation was observed for the non-encapsulated AA. In the first 60 min of the experiment, almost half of the AA present in solution had already been degraded, while no AA remained in the solution after 120 min, showing that the AA was totally degraded. For the NPs_CS_AA and NPs_PCL_AA, the AA degradation rates were slower, with 60.88 ± 0.05% and 67.15 ± 0.52% of the AA remaining after 60 min, and total degradation of AA after 360 and 480 min, respectively. These results demonstrated the effectiveness of the nanosystems in protecting AA against degradation and corroborated the data obtained using the peroxymonosulfate oxidizing agent. In addition, these results showed that under aquarium conditions, the use of the nanoparticles could promote an increase in the amount of AA in solution, compared to non-encapsulated AA. The results obtained in these tests corroborated the release data, with the greater degradation observed for NPs_CS_AA being due to the faster release of the compound, compared to the release from NPs_PCL_AA, with the latter promoting a more sustained release of the AA protected within the nanoparticles.

AA solutions are highly susceptible to rapid degradation and oxidation by oxygen dissolved in alkaline solutions [36]. Stevanovic et al*.* [37] prepared poly(D,L-lactide-co-glycolide) nanospheres for the encapsulation of ascorbic acid and evaluated the in vitro degradation of the particles in the presence and absence of ascorbic acid. In both cases, degradation and complete release of the ascorbic acid occurred after 8 weeks. Only 10% of the ascorbic acid was released in the first 24 days, after which agglomeration of the particles resulted in the formation of a porous film. The encapsulation of AA in the nanoparticles provided protection against reactions, temperature, humidity, and oxygen, consequently improving both stability and bioavailability.

The degradation of AA is influenced by factors including oxygen, pH, temperature, metal ions, enzymes, light, water activity, and oxidizing agents (nitrite), among others [38]. Studies have mainly focused on the degradation of ascorbic acid in fruits and other foods, which generally follows first order kinetics [39]. When oxygen is present, the rate of degradation of AA is faster [40]. This work is the first study of the oxidation of AA (99% purity) encapsulated in polymeric nanoparticles, comparing the results obtained with induction using peroxymonosulfate and under aquarium conditions, which opens up new opportunities for future research.

### Toxicity evaluation using zebrafish

**Fig. 4:**
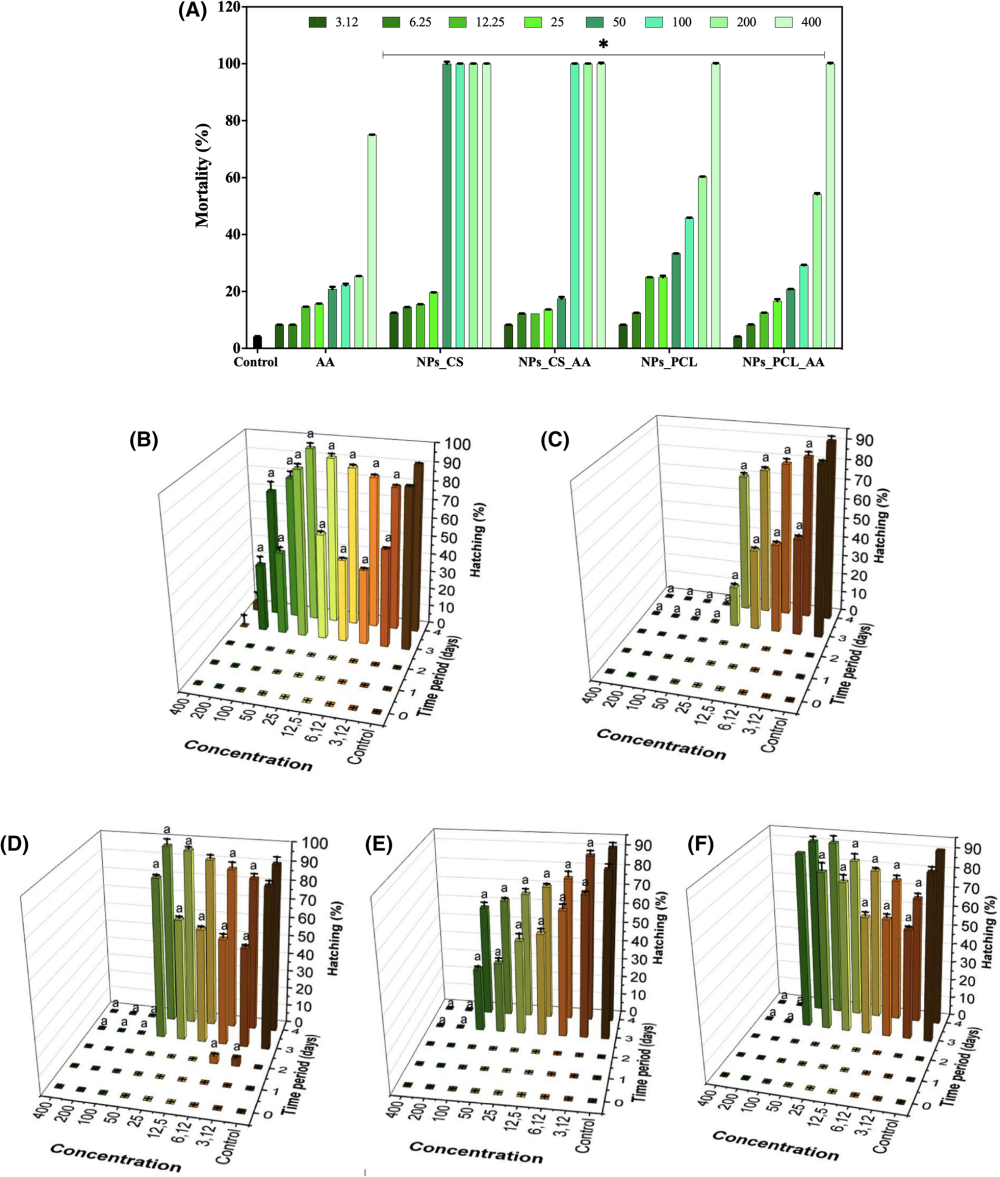
**A** Mortality rates of embryos and larvae exposed to the different treatments (control, AA, NPs_CS, NPs_CS_AA, NPs_PCL, and NPs_PCL_AA) at different concentrations (3.12, 6.25, 12.25, 25, 50, 100, 200, and 400 mg/L). **B–F** Hatching rates of embryos exposed to ascorbic acid (AA), chitosan nanoparticles (NPs_CS), chitosan nanoparticles containing AA (NPs_CS_AA), PCL nanoparticles (NPs_PCL), and PCL nanoparticles containing AA (NPs_PCL_AA). For the statistical tests, ANOVA (two-way, significance of p < 0.05) was used to observe the statistical differences between groups with the same concentration, where a indicates a statistically significant difference, compared to the control group

The use of nanomaterials has grown exponentially, accompanied by increasing concerns about their toxicities due to occasional or occupational exposures. Several factors can influence the toxicity of the materials, both intrinsic to them (size and shape, surface charge, composition, and state of agglomeration, among others) and extrinsic (such as pH, ionic strength, and medium composition). In this work, the LC_50_ values were determined for non-encapsulated AA, chitosan nanoparticles, chitosan nanoparticles containing AA, PCL nanoparticles, and PLC nanoparticles containing AA. Zebrafish embryos were used as a model organism, following the OECD 236 protocol (OECD, 2013). The concentrations used were 3.12, 6.25, 12.5, 25, 50, 100, 200, and 400 mg L^−1^. The accumulated mortality percentage was measured after 96 h (Fig. 4A).

The groups treated with non-encapsulated AA showed mortality rates below 30% for all the concentrations tested, with the exception of the highest concentration (400 mg L^−1^), where mortality was 75% (Fig. 4A), which could have been due to the pro-oxidant effect of AA at high concentrations, causing oxidative damage to DNA [41].

When AA was encapsulated in the chitosan NPs (NPs_CS_AA), mortality of less than 20% was observed up to a concentration of 50 mg L^−1^, while higher concentrations caused 100% mortality of the organisms. Chitosan nanoparticles without encapsulated AA (NPs_CS) were also tested at the same dilutions as the NPs containing AA (Fig. 4A). These formulations were more toxic to the zebrafish, with a concentration of 50 mg L^−1^ causing 100% mortality of the organisms. The zebrafish treated with PCL nanoparticles (NPs_PCL) and PCL nanoparticles containing AA (NPs_PCL_AA) showed concentration-dependent mortality. In both cases, mortality of less than 50% was observed up to a concentration of 100 mg L^−1^. However, the highest concentration (400 mg/L) caused 100% mortality.

The mortality data indicated that the chitosan nanoparticles were more toxic to the zebrafish embryos and larvae, compared to the PCL nanoparticles. Probit analysis, performed with Statgraphics Centurion XVII v. 1.17.04 software, enabled calculation of the LC_50,_ LC_40_, LC_30_, LC_20_, and LC_10_ values for the different formulations tested (Table 2).

**Table 2:** Average lethal concentrations (mg/L) for zebrafish embryos and larvae exposed to different treatments: ascorbic acid (AA), chitosan nanoparticles (NPs_CS), chitosan nanoparticles containing ascorbic acid (NPs_CS_AA), PCL nanoparticles (NPs_PCL), and PCL nanoparticles containing AA (NPs_PCL_AA)

LC – 96 h (mg/L)	AA	NPs_CS	NPs_CS_AA	NPs_PCL	NPs_PCL_AA
LC_50_	330.7	28.9	57.4	168.9	179.6
LC_40_	275.8	24.8	48.1	137.5	146.1
LC_30_	217.1	20.3	38.1	103.8	110.2
LC_20_	148.3	15.2	26.4	64.5	68.2
LC_10_	53.0	8.0	10.2	9.9	10.0

The concentrations tested for each exposure were LC_10_, LC_20_, LC_30_, LC_40_, and LC_50_. The LC values after 96 h of exposure were determined by probit analysis, employing Statgraphics Centurion XVII v. 1.17.04 software (Stat Point Technologies, 2014). The plates were analyzed using a stereomicroscope (Model SMZ 2 LED, Optika), at 2 × magnification

The LC_50_ for non-encapsulated AA was 330.7 mg L^−1^, while reductions of 11.4-fold and 5.7-fold, respectively, were observed for the LC_50_ values of the chitosan nanoparticles (28.9 mg L^−1^) and chitosan nanoparticles containing AA (57.4 mg/L), compared to the non-encapsulated AA. The LC_50_ values for the NPs_PCL and NPs_PCL_AA formulations were 168.9 and 179.6 mg L^−1^, respectively, around twofold lower than the LC_50_ for the non-encapsulated AA.

The LC_50_ was significantly higher after the encapsulation of AA in the chitosan nanoparticles (NPs_CS_AA), compared to the nanoparticles alone (NPs_CS). This increase could have been related to the ability of ascorbic acid to modulate or decrease genotoxicity, regenerating other oxidants and removing free radicals.

Similar results were obtained elsewhere for the LC_50_ of chitosan nanoparticles for zebrafish embryos (LC_ß_ = 23.26 mg L^−1^) [42]. In this case, the encapsulated ascorbic acid acted to reduce the toxic effects of the chitosan particles [41].

The incorporation of AA into the NPs_PCL system also decreased the mortality rate, which could be attributed to the effect of AA in reducing toxicity. This effect was reported by Stevanović et al*.* [43], who found that the incorporation of ascorbic acid significantly reduced genotoxicity in *Oreochromis mossambicus* exposed to ethyl methanesulfonate. Sarma et al*.* [44] found that the incorporation of ascorbic acid in the diet reduced the toxicity of the acaricide endosulfan, tested using *Channa punctatus*. The antioxidant activity of AA can act to decrease the toxicity of certain compounds. However, the observed behavior may have been more related to the release of AA from the NPs.

The toxicity of PCL nanoparticles has been evaluated in other in vitro and in vivo studies. Quercetin-loaded polycaprolactone microspheres were evaluated for their in vitro cytotoxicity in synovial cells [45]. The treatments were carried out for 48 h, in the presence and absence of the active substance (quercetin). The results showed that the PCL microspheres caused cytotoxicity in the cells, suggesting that the system could be used in future treatment methodologies. In other work, Youssouf et al*.* [46] tested the effects of oligo-carrageenan micelles grafted with polycaprolactone, containing curcumin. It was found that the micelles were not cytotoxic in zebrafish and that they also decreased the anti-inflammatory effects of curcumin and improved cell absorption. It was concluded that the micelles were safe nanocarriers.

In addition to mortality, evaluation was made of the influence of the formulations on the hatching of zebrafish. The embryos were exposed to non-encapsulated AA, chitosan NPs (NPs_CS), chitosan NPs containing AA (NPs_CS_AA), PCL NPs (NPs_PCL), and PCL nanoparticles containing AA (NPs_PCL_AA), at concentrations of up to 200, 25, 50, and 100 mg L^−1^, respectively. The concentrations were defined according to the mortality tests and concentrations higher than the LC_50_ of each formulation were not used.

The hatching rates of the embryos were lower after 48 h of exposure to AA, compared to the control group (Fig. 4B). However, after 96 h, the hatching rate of the embryos exposed to a concentration of 50 mg/L was similar to that of the control group. For concentrations of 100 and 200 mg L^−1^, more pronounced decreases in hatching were observed after 96 h of treatment. Lower hatching rates were also observed for the embryos exposed to the chitosan NPs (Fig. 4C). However, for these groups, the hatching decreased in a dose-dependent manner, according to the increase in the concentration of nanoparticles. Interestingly, when the embryos were exposed to nanoparticles loaded with AA, the hatching rate was also lower, compared to the control group, but hatching increased with increase of the concentration of nanoparticles, after 96 h of exposure (Fig. 4D). For the embryos treated with the PCL NPs, the hatching rate was inversely proportional to the concentration of nanoparticles present in the medium, with a higher concentration of NPs resulting in a lower hatching rate (Fig. 4E). All the concentrations tested caused a significant reduction in the hatching rate after 96 h of exposure, with the exception of the lowest concentration tested (3.12 mg L^−1^). For the embryos exposed to the PCL NPs containing AA, as well as those exposed to the chitosan nanoparticles, the hatching rate increased with increase of the concentration of NPs in the medium (Fig. 4F).

Similar results were reported by Hu et al*.* [47], who evaluated the toxicity of chitosan nanoparticles and chitosan nanoparticles containing Tween 80, with average sizes of 247 ± 20 and 251 ± 15 nm, respectively, using zebrafish as a model organism. Dose-dependent decrease of the hatching rate and increase of mortality were observed for both formulations, with an embryo mortality rate of around 100% at a concentration of 50 mg/L. The LC_50_ values for the chitosan nanoparticles and the chitosan nanoparticles containing Tween were 23.26 and 25.06 mg L^−1^, respectively, which were very similar to those found in this study (Table 2).

Hu et al*.* [47] found that chitosan nanoparticles with sizes of 200 and 340 nm caused significant mortality of zebrafish embryos, compared to the positive control (ZnO nanoparticles), at concentrations of 40 and 30 mg L^−1^, respectively. For both types of nanoparticle, the toxicity to the embryos was dose- and time-dependent, with the smaller nanoparticles having greater toxic effects.

In contrast, Wang et al*.* [48] tested the toxicity of chitosan nanoparticles (84.86 nm) towards zebrafish embryos, using different concentrations (150 to 400 mg L^−1^), obtaining an LC_50_ approximately tenfold higher (280 mg L^−1^) than found in the present study. In another zebrafish toxicity study, Abou-Saleh et al*.* [49] found that chitosan nanoparticles in the size range 100–150 nm presented no toxic effects until the highest concentration tested (200 mg L^−1^). These differences between the studies suggest the need for further research investigating the effects of different preparation methods, since it is possible that specific components of the nanoparticles could have induced toxic effects in the zebrafish embryos.

Evaluation was also made of morphological changes in the zebrafish embryos and larvae (Additional file 1: Figure S1). For all groups exposed to low concentrations, no abnormalities were observed in the embryos and larvae. However, high concentrations caused toxic effects including edema and opacity of the yolk sac, tail malformation, clotted egg, change in head formation, and disintegration of larvae. The non-encapsulated AA caused effects at concentrations of 25 and 50 mg L^−1^, with the organisms showing higher numbers of malformations, compared to the organisms exposed to AA encapsulated in the NPs_CS_AA, at the same concentrations. After exposure to 100 mg L^−1^ of NPs_ CS_ AA, the larvae disintegrated after hatching. The embryos showed changes in the formation of the head and were coagulated when exposed to NPs_CS concentrations of 50 mg L^−1^ or higher (Additional file 1: Figure S1).

Malformations were also observed in the animals treated with PCL nanoparticles and PCL nanoparticles containing AA. However, at the same concentration (100 mg L^−1^), fewer types of malformations were observed in the animals exposed to NP_PCL_AA, compared to non-encapsulated AA (Additional file 1: Figure S1). It should be noted that ascorbic acid (AA) is a vitamin and has limited toxicity, although high concentrations (above 200 mg L^−1^) can cause some toxic effects. The results showed that the chitosan nanoparticles had toxic effects in the embryos, but were less toxic when combined with ascorbic acid, which led to fewer morphological abnormalities and a higher hatching rate, due to the controlled release of AA from the NPs.

The results indicated that concentrations of up to 50 and 100 mg L^−1^ of AA encapsulated in NPs_CS_AA and NPs_PCL_AA, respectively, would be promising for the incorporation of these systems as a vehicle for this vitamin in fish feed. These doses are higher than those necessary for the satisfactory development of fish [50, 51]. For the incorporation of AA in the feed, advantages of the systems proposed here are that they can reduce oxidation of the compound and prolong its shelf life, due to the high encapsulation efficiency (above 90%, for both nanoparticulate systems), providing stability during 90 days of storage (data not shown).

### Evaluation of fish embryo acute toxicity (FET)

**Fig. 5:**
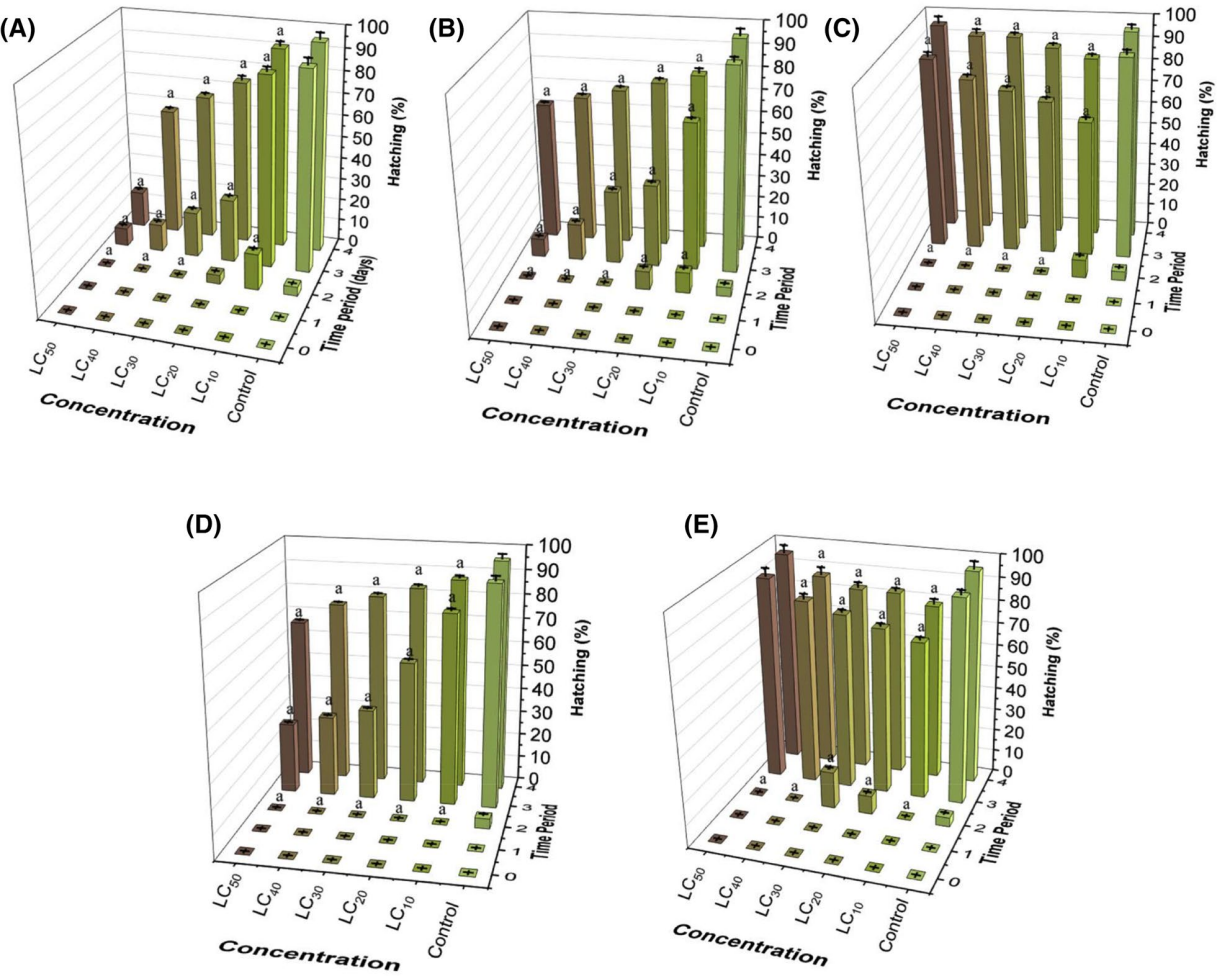
Hatching rates of embryos and larvae exposed to the treatments at different concentrations (LC_10_, LC_20_, LC_30_, LC_40_, and LC_50_): **A** ascorbic acid (AA), **B** chitosan nanoparticles (NPs_CS), **C** chitosan nanoparticles containing AA (NPs_CS_AA), **D** PCL nanoparticles (NPs_PCL), and **E** PCL nanoparticles containing AA (NPs_PCL_AA). For the statistical tests, ANOVA (two-way, significance of p < 0.05) was used to identify differences between the groups, where a indicates a statistically significant difference, compared to the control group

The morphological changes (Additional file 1: Figure S2) of the embryos and larvae were evaluated for 96 h exposure to different concentrations (LC_10_, LC_20_, LC_30_, LC_40_, and LC_50_) of all the formulations. After fertilization, the embryos were individually exposed in 24-well polystyrene plates kept in an incubator at 26 ± 1 °C. The lethal effect on the embryos was indicated by the absence of somites, egg coagulation, and loss of independent movement. Hatching was used to assess toxicity, since it is influenced by several environmental and endogenous factors. Figure 5 shows the daily rate of embryo hatching. For all treatments, at the lowest concentrations tested (LC_10_ and LC_20_), hatching started after 48 h of exposure, while at higher concentrations, hatching started after 72 h of exposure. For embryos treated with non-encapsulated AA, hatching decreased in a dose-dependent manner, with only 16.6% of the embryos hatching after 96 h of exposure at the highest concentration tested (LC_50_) (Fig. 5A). Although ascorbic acid has an antioxidant effect, at high concentrations it can act as a pro-oxidant, causing oxidative damage to DNA [41].

The embryos treated with PCL nanoparticles at the LC_50_ concentration showed a hatching rate of 66.7% after exposure for 96 h, which was 1.4-fold lower than observed for the control group after the same exposure time. In contrast, the hatching rate values for the animals treated with NPs_PCL_AA were higher than 79% for all the concentrations tested. In the case of NPs_CS_AA, the hatching rate increased with increase of the concentration of AA present in the medium. For the animals exposed to the LC_50_ concentration, the hatching rate after 96 h was 95.8%, which was the same as the value observed for the control after 96 h. These results indicated that the sustained release of AA acted to reduce the effects of the nanoparticles on the hatching of embryos, since in the presence of AA there was no significant difference for this parameter, when compared to the control group.

Embryo malformation was assessed using images obtained with a stereomicroscope (Model SMZ 2 LED, Optika), at 2 × magnification. Additional file 1: Figure S2 shows the malformations of embryos and larvae observed for each treatment at the LC_50_ dose, after 96 h of exposure. Regardless of the treatment, the embryos presented malformation of the yolk sac. However, the embryos exposed to non-encapsulated AA showed greater severity and number of abnormalities such as edema and opacity of the yolk sac, edema of the pericardium, malformation of the tail, and bent spine.

All these teratogenic alterations, with the exception of tail malformation, were also observed for the embryos treated with chitosan nanoparticles. Embryos exposed to the PCL nanoparticles showed malformation, edema, and opacity of the yolk sac, as well as pericardial edema. These same malformations, except pericardial edema, were observed for the embryos treated with PCL nanoparticles containing AA.

Hu et al*.* [47] also observed dose-dependent malformations including bent spine and opaque yolk, following exposure of the organisms to chitosan nanoparticles (mean diameter of 200 nm). However, no significant malformations were observed in embryos exposed to chitosan nanoparticles with mean diameter of 340 nm. Yuan et al*.* [42] observed non-inflated swim bladder and bent spine in embryos exposed to both chitosan and chitosan/Tween 80 nanoparticles.

The embryos treated with nanoparticles containing AA showed only edema and opacity of the yolk sac. Encapsulation of the AA led to decreased toxicity, evidenced by fewer malformations, compared to the non-encapsulated compound and the control nanoparticles. These results suggested that the interaction between ascorbic acid and nanoparticles could decrease the toxic effect of the carrier system, while providing efficient release of the active compound.

### Evaluation of zebrafish motor activity

The use of zebrafish as a model for behavioral studies opens up new possibilities for assessing the toxicity of nanoparticles. In order to identify the effects of the treatments on motor development, evaluation was made of the speed of the zebrafish larvae and the distance traveled. Prolonged exposure to stressors can generate an adaptation response, consequently affecting the health of the body. When exposed to harmful compounds, fish may show stress responses such as slowing down, in order to save energy, or adoption of avoidance behavior, in order to increase the chance of survival [52].

**Fig. 6:**
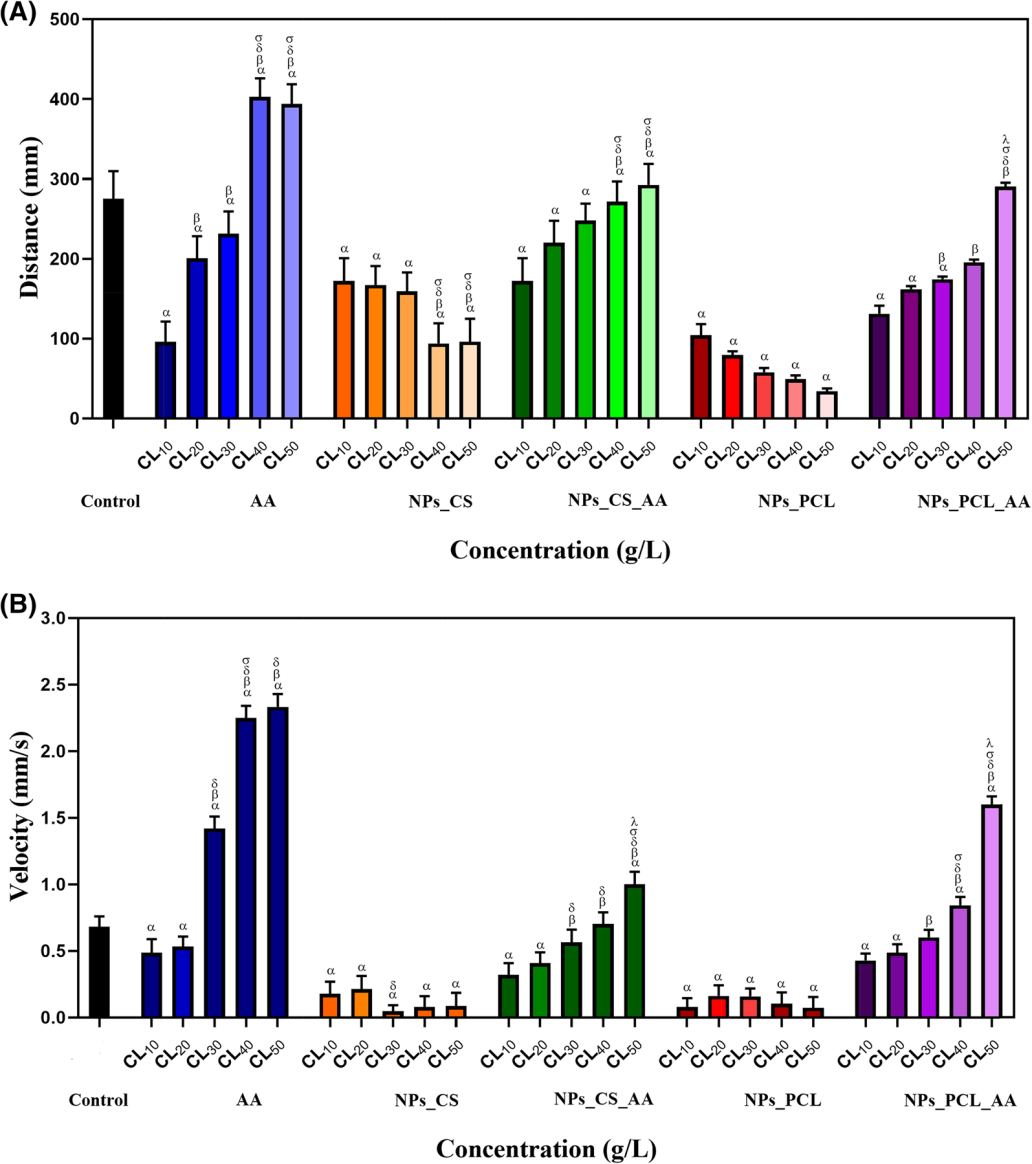
Evaluation of the motor development biomarkers **A** distance traveled and **B** speed for zebrafish (*Danio rerio*) larvae exposed to the different treatments: control, ascorbic acid (AA), CS nanoparticles (NPs_CS), CS nanoparticles containing ascorbic acid (NPs_CS_AA), PCL nanoparticles (NPs_PCL), and PCL nanoparticles containing ascorbic acid (NPs_PCL_AA). The exposures were performed for 96 h at different concentrations (LC_10_, LC_20_, LC_30_, LC_40_, and LC_50_). For the statistical tests, ANOVA (two-way, significance of p < 0.05) was used to evaluate differences among the groups, with α, β, δ, σ, and λ indicating statistically significant differences from the control, LC_10_, LC_20_, LC_30_, and LC_40_ groups, respectively (n = 9)

Analysis was made of the behavioral biomarkers (speed and distance covered) for larvae exposed to different concentrations (LC_10_, LC_20_, LC_30_, LC_40_, and LC_50_) of the formulations (non-encapsulated AA, chitosan nanoparticles (NPs_CS), chitosan nanoparticles containing AA (NPs_CS_AA), PCL nanoparticles (NPs_PCL), and PCL nanoparticles containing AA (NPs_PCL_AA). The embryos were exposed for 96 h, with subsequent evaluation of distance and speed using EthovisionXT software (Fig. 6).

Both the larvae exposed to non-encapsulated AA and the larvae exposed to chitosan nanoparticles containing AA showed a dose-dependent increase in the distance traveled, with higher concentrations resulting in greater distances traveled (Fig. 6A). Compared to the control group, the distance traveled was shorter, up to the LC_30_ concentration, for the animals exposed to AA and those exposed to NPs_CS_AA. However, for non-encapsulated AA at the LC_50_, there was a significant (p < 0.05) increase in the distance traveled, compared to the control group, whereas use of the encapsulated ascorbic acid (in both types of nanoparticle) at the LC_50_ did not result in a significant difference in the distance traveled, compared to the control. The larvae exposed to the chitosan and PCL nanoparticles (without ascorbic acid) presented dose-dependent reductions in the distance traveled, compared to the control group.

The swimming speed behavior was similar to that for the distance covered. Larvae treated with non-encapsulated AA and AA encapsulated in chitosan and PCL nanoparticles showed dose-dependent increases in speed, while larvae treated with chitosan and PCL nanoparticles (without ascorbic acid) presented decreased swimming speed with increased concentration of nanoparticles in the medium (Fig. 6B). There is a known relationship between speed and distance traveled, where the increased swimming activity may be driven by the escape instinct or the response to stress [53].

The external integument or skin is a form of protection for fish, with the mucosa and scales playing important roles in protecting against pathogens, in addition to providing osmoregulatory and respiratory functions [54, 55]. The motor behavior inhibition caused by the chitosan and PCL NPs could have been due to the mucoadhesiveness of the nanoformulations, since fish epithelial cells are coated with a mucus layer rich in mucopolysaccharides and mucoproteins, enabling nanoparticles to adhere to the gills and skin [16]. The absence of the antioxidant effect of AA may have influenced the results, since for both types of nanoparticle containing AA, the locomotion activity gradually increased with increase of the AA concentration. Larvae exposed to the LC_40_ and LC_50_ concentrations of both systems (NPs_CS_AA and NPs_PCL_AA) were more active, compared to the control group.

However, different to the present findings, previous studies found that the treatment of embryos with chitosan nanoparticles caused an abnormal increase in locomotion activity (hyperactivity), compared to the control group [42, 49]. It was suggested that the chitosan nanoparticles were able to negatively affect the embryo nervous system, but did not affect muscular development. As noted by Wong et al*.* [56], anomalies observed in locomotion can be caused by changes in neuromuscular function and problems with neuronal innervations or muscles.

The increases in activity of the AA, NPs_CS_AA, and NPs_PCL_AA groups could be related to the electrical signals of primary motor neurons, on which tail movement and increased swimming depend [57]. Primary motor neurons can periodically or spontaneously depolarize [58], with the frequency of this depolarization being related to the behavior of the fish, whereby increases in the electrical signals and muscle contraction lead to increases of speed and the distance traveled. For the organisms exposed to NPs_CS and NPs_PCL, the locomotion activity was lower in comparison with all the other groups tested, with no significant differences between the treatments. It is likely that the larvae were in a process of habituation, defined as a decrease in the conduct response, due to sensory or motor fatigue [59].

For both types of nanoparticle containing AA, the locomotion activity gradually increased with increase of the AA concentration, with the LC_50_ concentration causing the larvae to become more active than the organisms in the control group. In addition to these factors, the distance traveled by the zebrafish larvae was also related to the energy reserve provided by the yolk sac or yolk vesicle, which stores a nutrient substance (calf) responsible for providing the embryo with nutrients from the beginning of development until the start of exogenous feeding by the larva [60]. The loss of nutrients or failures in the absorption of nutrients from the yolk sac can result in decreased embryonic growth, due to decreased blood flow and cardiac changes [61–63]. Other changes observed include pericardial edema, yolk sac edema, opaque yolk, tail malformation, and bent spine.

### *Evaluation of acetylcholinesterase (AChE)activity*

In the areas of neuropharmacology and neurotoxicology, zebrafish provides a very useful model for study of the effects of different compounds, enabling investigation of mechanisms of action as well as functional effects, such as behavioral changes. Swimming behavior can be used as a sensitive endpoint for detection of the sublethal effects of pollutants in fish [64].

**Fig. 7:**
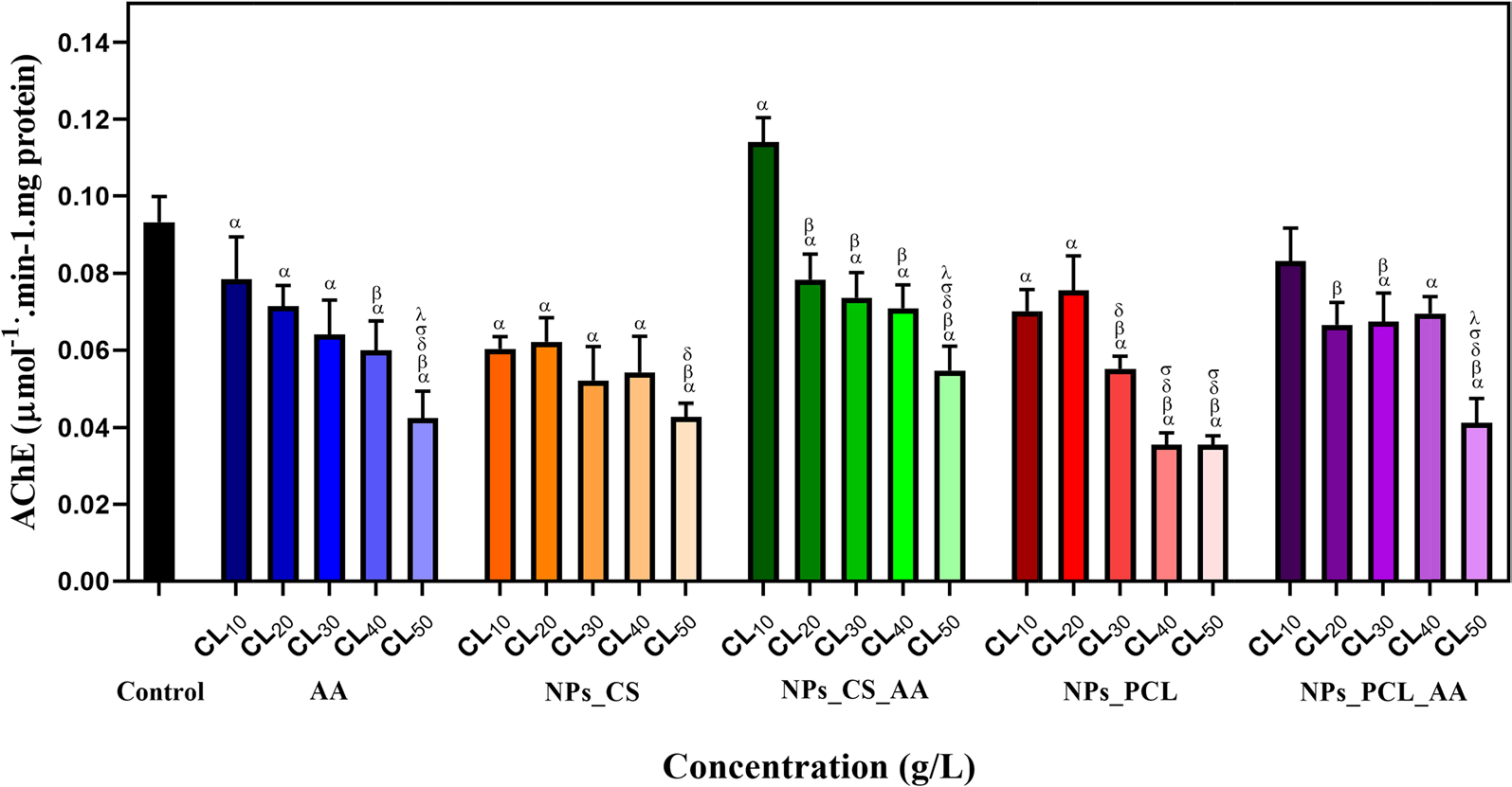
Acetylcholinesterase (AChE) activities of zebrafish larvae after exposure for 96 h to different concentrations (LC_10_, LC_20_, LC_30_, LC_40_, and LC_50_) of ascorbic acid (AA), CS nanoparticles (NPs_CS), CS nanoparticles containing AA (NPs_CS_AA), PCL nanoparticles (NPs_PCL), and PCL nanoparticles containing AA (NPs_PCL_AA), compared to the control. For the statistical tests, Kruskal-Wallis test (p < 0.05) followed by Dun test was used to evaluate differences among the groups, with α, β, δ, σ, and λ indicating statistically significant differences from the control, LC_10_, LC_20_, LC_30_, and LC_40_ groups, respectively (n = 9)

The specific AChE activity was determined for the larvae exposed to the different treatments (non-encapsulated AA, chitosan nanoparticles (NPs_CS), chitosan nanoparticles containing AA (NPs_CS_AA), PCL nanoparticles (NPs_PCL), and PCL nanoparticles containing AA (NPs_PCL_AA)) at different concentrations (LC_10_, LC_20,_ LC_30_, LC_40_, and LC_50_). Statistical analysis employed the Kruskal–Wallis test (p = 0.05) followed by the Dunn test (Fig. 7).

Most of the treatments caused a significant dose-dependent decrease in AChE activity, compared to the negative control. An exception was the chitosan nanoparticles containing encapsulated AA, at the lowest concentration tested (LC_10_), where the activity of this enzyme was significantly higher, compared to the control group. Wong et al*.* [65] showed that the inhibition of the AChE enzyme by nanoparticles can be caused by its adsorption or interaction with the particles. It was observed that following adsorption, the nanoparticles inhibited AChE differently, depending on their chemical composition.

ACh plays a crucial role in the central and neuromuscular synapses of the cholinergic system. When ACh is released into the synaptic cleft, it is rapidly degraded by AchE [66]. Since AChE is the enzyme responsible for ending cholinergic activity in the synaptic cleft, AChE inhibition leads to accumulation of the neurotransmitter acetylcholine (ACh), resulting in increased stimulation of cholinergic neurons. Consequently, inhibition of the AChE enzyme can affect the balance and locomotion of the exposed animals. Excessive hydrolysis of ACh by AChE may lead to muscle hypoactivity and decreased behavioral responses [67]. In severe cases, it can also cause collapse of the central nervous system, resulting in loss of muscle control or even death [68, 69]. Also, the cholinergic system may be involved in morphometric alterations such as curvature of the fish tail, due to abnormal muscle development caused by inhibition of acetylcholinesterase activity and the lack of hydrolysis of AChE [70, 71]. Hence, it is important to establish the relationship between locomotion behavior and biochemical responses such as AChE activity [21].

Alteration of this enzyme activity can lead to motor function impairment in zebrafish, due to the change in ACh levels at the neuromuscular junction [21, 72]. Behavioral impairment of silver catfish has been attributed to inhibition of cerebral AChE activity and oxidative damage [73]. Similarly, Baldissera et al*.* [74] suggested that exposure to aquaculture-relevant concentrations (11 mg L^−1^) of trichlorfon, an organophosphate pesticide, could elicit behavioral abnormalities associated with impairment of locomotion activity, a condition that can be caused by cerebral oxidative damage, depression of the antioxidant defense system, and inhibition of AChE activity [75]. Studies have suggested the existence of a threshold relationship between AChE inhibition and swimming performance. Sandahl et al*.* [76] observed significant decreases in swimming activity at an exposure concentration of chlorpyrifos as low as 0.6 µg L^−1^, corresponding to a relative AChE activity of 77.2%, compared to the control.

Tierney et al. [77] suggested that the AChE inhibition threshold was determined by the type of muscle used for any given swimming modality (swimming powered by aerobic muscle, or swimming powered anaerobically, depending on the difference in the amounts of red and white tissues). Critical swimming capacity may remain available, even when AChE inhibition reduces spontaneous swimming activity. This suggests that any anticholinesterase exposure impairing spontaneous swimming, a swimming mode that enables foraging, may still leave fish with maximum swimming performance, leading to a proportional increase in the use of white muscle, which may differentially impair swimming behaviors such as predator avoidance.

However, for these observed effects, it can be difficult to distinguish between cholinergic (central and peripheral) mechanisms and other systems that participate in the processes of locomotion and swimming. It is interesting that two organophosphate pesticides (chlorpyrifos and malathion) were found to cause opposing behaviors in zebrafish larvae, related to swim speed (hyperactivity *vs.* hypoactivity) and rest. The swim speed and rest behaviors appeared to be correlated to AChE activity in larvae treated with chlorpyrifos, but not in larvae treated with malathion [78]. The findings suggest that there may be other neural parameters that are affected and that influence larval behavioral changes. Gene modulation may be involved in cholinergic neurotransmission [79], while pharmacological and optogenetic manipulations of cholinergic neurotransmission in the hippocampal and amygdala regions suggest a specific role of cholinergic receptors in modulating the conditioned fear and extinction responses [75]. Furthermore, in rapid acceleration movements, red muscle not only produces power, but also has an important role in tuning the effective body stiffness. The ongoing activity of red muscle may be a consequence of the motor neuron recruitment pattern for red and white muscle, with motor neurons for red muscle being recruited earlier, due to their low resistance, and remaining activated even when motor neurons for white muscle are recruited [80].

It is also interesting to note that ascorbic acid plays a role in neuronal differentiation, maturation, myelin formation, and modulation of neurotransmission, as in cholinergic systems [81]. Based on these results, it is plausible that AA interferes with the cholinergic system, depending on the dose and possibly the fish stage of development. Although, in the present study, a decrease in AchE levels was observed, Narra et al*.* [82] showed that the acetylcholinesterase activity of rockfish was significantly increased by dietary AA supplementation, mitigating chlorpyrifos toxicity. In addition, dietary AA supplementation in the rockfish induced an increase in AChE activity [83]. Hence, it is not yet clear whether chitosan can affect the role of AA in development of the cholinergic neurotransmitter system, since chitosan nanoparticles can remain adhered to the mucosal surfaces of fish [16]. Another possibility is that the AA kinetics induced by nanoparticles, at the larval development stage selected here, might influence the enzyme activity at maturity, resulting in a non-synergistic effect.

Since locomotion behavior in organisms is important for avoiding predators, seeking food, and reproduction, it is a parameter of considerable ecological relevance [80, 84]. In the present work, the nanoformulations did not appear to negatively affect swimming performance, despite the AChE decrease. On the contrary, the velocity and distance covered by the zebrafish larvae increased, in a response evoked to assist the fish in escaping from predators. Future studies will be needed to further elucidate these effects in zebrafish larvae.

### Evaluation of zebrafish body weight

The control of fish nutrition is essential in the aquaculture industry, in order to maintain good health, strong immune systems, and resistance to disease [85]. The use of natural vitamins in aquaculture has been shown to provide good growth, satisfactory development, and normal cell function in aquatic organisms [86].

**Fig. 8:**
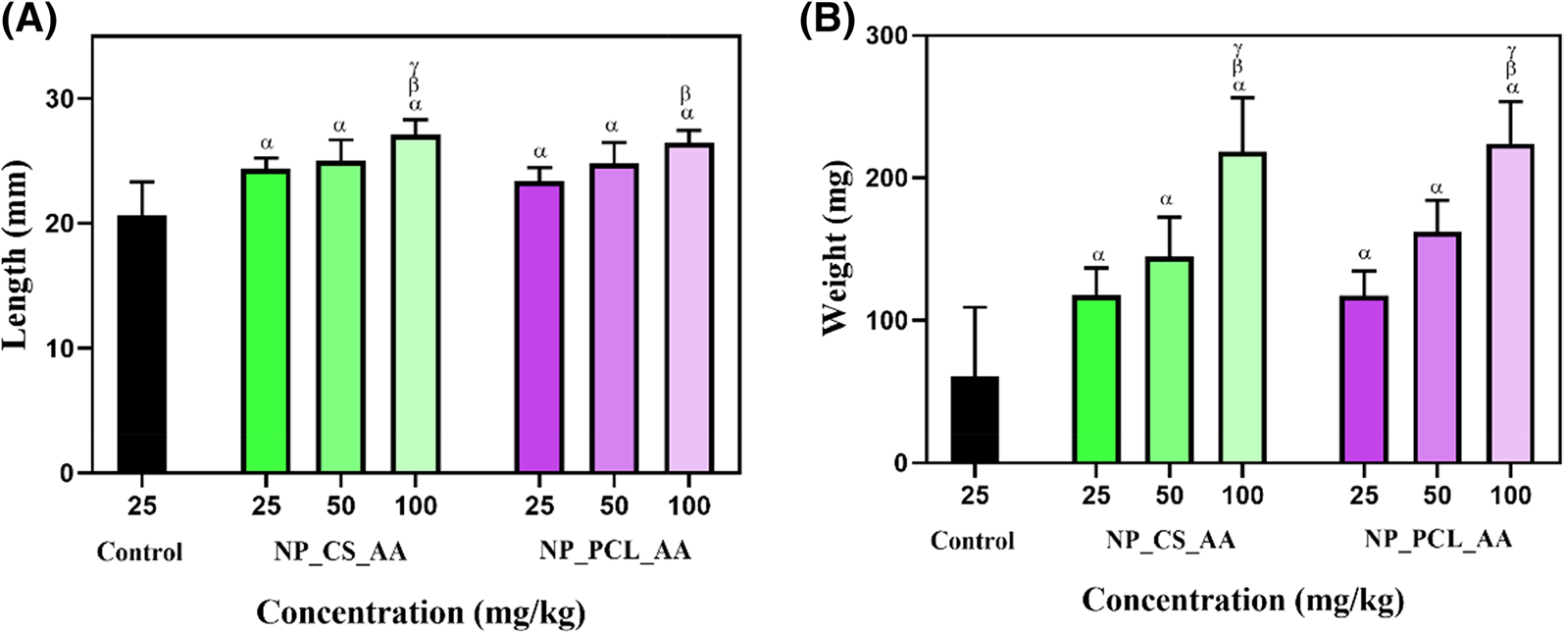
Evaluation of body length **A** and weight **B** of zebrafish fed different diets. The control group was fed commercial food (TetraMIN), while the other groups were fed TetraMIN supplemented with different concentrations (25, 50, and 100 mg/kg) of NPs_CS_AA and NPs_PCL_AA. Statistical tests were performed using ANOVA (two-way, significance of p < 0.05) to evaluate differences among the groups, where α, β, and δ indicate statistically significant differences from the control, 25 mg/kg, and 50 mg/kg groups, respectively (n = 10)

Ascorbic acid encapsulated in the CS and PCL nanoparticles was incorporated at different concentrations (25, 50, and 100 mg/kg) in a commercial fish feed (TetraMIN). Figure 8A shows the fish sizes obtained for the control group (TetraMIN alone) and the nanoparticle groups. The fish in the control group showed an average size of 20 mm, while the fish that received the commercial feed plus PCL nanoparticles (NPs_PCL_AA) at different concentrations showed a larger size of 23 ± 1.1 mm, representing an increase of 15%, compared to the control group. Similar results were obtained for the fish in the groups that received the commercial feed plus CS nanoparticles (NPs_CS_AA) at different concentrations, with an average size of 24 ± 0.8 mm, representing an increase of 20%, compared to the control group. The results showed that the higher the concentration of AA added to the food, the greater the size of the fish. Both groups treated with 100 mg/kg showed a significant difference in relation to the control group and the other concentrations, whereas there was no significant difference in fish size between the NPs_CS_AA and NPs_PCL_AA groups. These results indicated that neither colloidal system affected growth of the fish.

Figure 8B shows the weights of the fish after exposure to the different treatments. An average final weight of 60.97 mg was obtained for the control group fed the commercial food (TetraMIN). The groups fed the commercial food with the addition of NPs_PCL_AA and NPs_CS_AA showed higher weights, compared to the control, with the weight increasing as the AA concentration was increased, similar to the effect observed for fish length. The final mean weights for the groups fed 25, 50, and 100 mg kg^−1^ of NPs_CS_AA were 117.5 ± 19.10, 144.5 ± 27.71, and 218.7 ± 48.3 mg, respectively. The corresponding values for the NPs_PCL_AA group were 117.4 ± 17.1, 162.32 ± 21.8, and 223.9 ± 29.55 mg, respectively. These results showed that the AA stimulated the development of the fish, with no apparent harm caused by the nanocarrier systems.

The effects of AA supplementation on fish growth were studied by Nsonga et al*.* [87], who quantitatively determined the dietary requirement of AA for the tilapia species, as well as the effect of this vitamin on fish growth. Different concentrations of AA (20, 40, 60, and 80 mg kg^−1^) were tested and it was found that supplementation with AA at 60 mg kg^−1^ in the feed increased weight gain and maintained good fish health. Supplementation at 80 mg kg^−1^ did not significantly increase fish development, compared to the 60 mg kg^−1^ dosage. In the present work, a supplementation dosage of 100 mg/kg had a greater effect on fish development, compared to the lower dosages tested (25 and 50 mg kg^−1^). In the work of Nsonga et al*.* [87], it is possible that AA degradation may have occurred, since the vitamin was not protected, which contributed to the absence of any difference between the effects of the dosages of 60 and 80 mg/kg. In the present case, the AA was protected, enabling it to be more effectively directed at the fish, consequently resulting in better fish development when increasing amounts of AA were added to the feed. In other work, supplementation of the fish diet with chitosan nanoparticles had positive effects on body weight, weight gain, and specific growth rate of grey mullet (*Liza ramada*) [88]. Similar results were observed for Nile tilapia [84–86, 89, 90], rainbow trout [91], silver carp [92], and loach [93] fed diets enriched with chitosan and/or chitosan nanoparticles. These positive effects of chitosan could be attributed to its ability to activate digestive enzymes, as well as to inhibit pathogenic bacteria, while activating beneficial ones [94].

Although dietary supplementation with micro-scale chitosan has shown positive results in the growth of some fish species, another study found that supplementation of the diet with micro-scale chitosan (at 5 g kg^−1^) did not lead to any significant difference in silver carp growth parameters, compared to control groups [92]. On the other hand, the authors showed that the animals supplied with a diet supplemented with chitosan nanoparticles (5 g kg^−1^) showed 29% greater weight gain, higher SGR, and better FCR, compared to the control and micro-scale chitosan groups [92]. These results could be explained by the higher absorption and bioavailability of chitosan nanoparticles in the fish gastrointestinal tract [95], as well as increased residence time of the nanoparticles, which increased the contact with the absorptive epithelium, consequently directly influencing fish growth [92, 96]. Greater growth of fish supplemented with chitosan nanoparticles, compared to those supplemented with micro-scale chitosan, has been observed for species including *Oreochromis nicoticus* [96, 97] and *Clarias gariepinus* [98]. However, it should be noted that for the same diet formulation, there were differences in the weight gains of the different species tested, which could have been related to the fish growth rate [99].

## Conclusions

The results of this work demonstrated that the chitosan and PCL nanocarrier systems could be effectively loaded with ascorbic acid. High encapsulation efficiencies were obtained for both systems, with values of 96 ± 0.5% for NPs_CS_AA and 91 ± 0.2% for NPs_PCL_AA, enabling protection of the vitamin against degradation. The formulations presented average nanoparticle diameters of 314 ± 3 and 303 ± 1 nm, respectively, and were stable during storage. In vitro studies showed that both systems provided sustained release of AA, while oxidation tests showed that the PCL nanoparticles provided longer protection. In toxicity tests, the NPs_PCL_AA formulation showed a lower mortality rate, compared to the chitosan nanoparticles formulation, with LC_50_ values of 57.4 and 179.6 mg mL^−1^ for NPS_CS_AA and NPs_PCL_AA, respectively. Encapsulation of the AA resulted in decreased malformations in the zebrafish embryos and larvae, compared to the non-encapsulated compound. The larvae exposed to AA encapsulated in the chitosan and PCL nanoparticles showed dose-dependent increases of speed and distance traveled. In contrast, most of the groups showed significant dose-dependent decreases of AChE activity, which did not seem sufficient to affect behavior, but could have led to morphological changes influenced by the cholinergic system, such as curvature of the fish tail. The findings showed that the PCL nanoparticles system had greater potential to protect ascorbic acid and also presented lower toxicity, compared to the chitosan nanoparticles system, possibly due to the slower release of the active compound from the PCL nanoparticles. The results demonstrated that the use of nanotechnology can open up new perspectives in aquaculture, helping to reduce feed nutrient losses, promoting faster growth of fish, and potentially reducing production costs. The use of nanotechnology in aquaculture can make an important contribution to the sustainable provision of healthy products in the food industry. The findings also demonstrated the suitability of zebrafish developmental parameters for use in environmental risk assessments of compounds and nanomaterials.

## Supplementary Information

**Additional file 1: Figure S1.** Morphological abnormalities (pericardial edema, skin lesions, undeveloped tail, tail alterations, and bent spine) observed in the zebrafish embryos and larvae exposed to the different treatments at concentrations of 25, 50, and 100 mg/mL: non-encapsulated ascorbic acid (AA), chitosan nanoparticles (NPs_CS), chitosan nanoparticles containing AA (NPs_CS_AA), PCL nanoparticles (NPs_PCL), and PCL nanoparticles containing AA (NPs_PCL_AA). The images were acquired using a stereomicroscope (Model SMZ 2 LED, Optika), at 2x magnification. **Figure S2.** Morphological abnormalities (yolk sac malformation and edema, pericardial edema, opaque yolk, tail malformation, and bent spine) observed in the zebrafish embryos and larvae exposed to the different treatments: non-encapsulated ascorbic acid (AA), chitosan nanoparticles (NPs_CS), chitosan nanoparticles containing AA (NPs_CS_AA), PCL nanoparticles (NPs_PCL), and PCL nanoparticles containing AA (NPs_PCL_AA). The images were acquired using a stereomicroscope (Model SMZ 2 LED, Optika), at 2x magnification.

## Data Availability

Not applicable.
