# Supplementary material for: Ecotoxicity evaluation of polymeric nanoparticles loaded with ascorbic acid for fish nutrition in aquaculture
Source: J Nanobiotechnology. 2021 May 31;19:163. doi: 10.1186/s12951-021-00910-8 (PMC8166143; doi:10.1186/s12951-021-00910-8)

**Additional file**

**Figure S1.** Morphological abnormalities (pericardial edema, skin lesions, undeveloped tail, tail alterations, and bent spine) observed in the zebrafish embryos and larvae exposed to the different treatments at concentrations of 25, 50, and 100 mg/mL: non-encapsulated ascorbic acid (AA), chitosan nanoparticles (NPs_CS), chitosan nanoparticles containing AA (NPs_CS_AA), PCL nanoparticles (NPs_PCL), and PCL nanoparticles containing AA (NPs_PCL_AA). The images were acquired using a stereomicroscope (Model SMZ 2 LED, Optika), at 2x magnification.


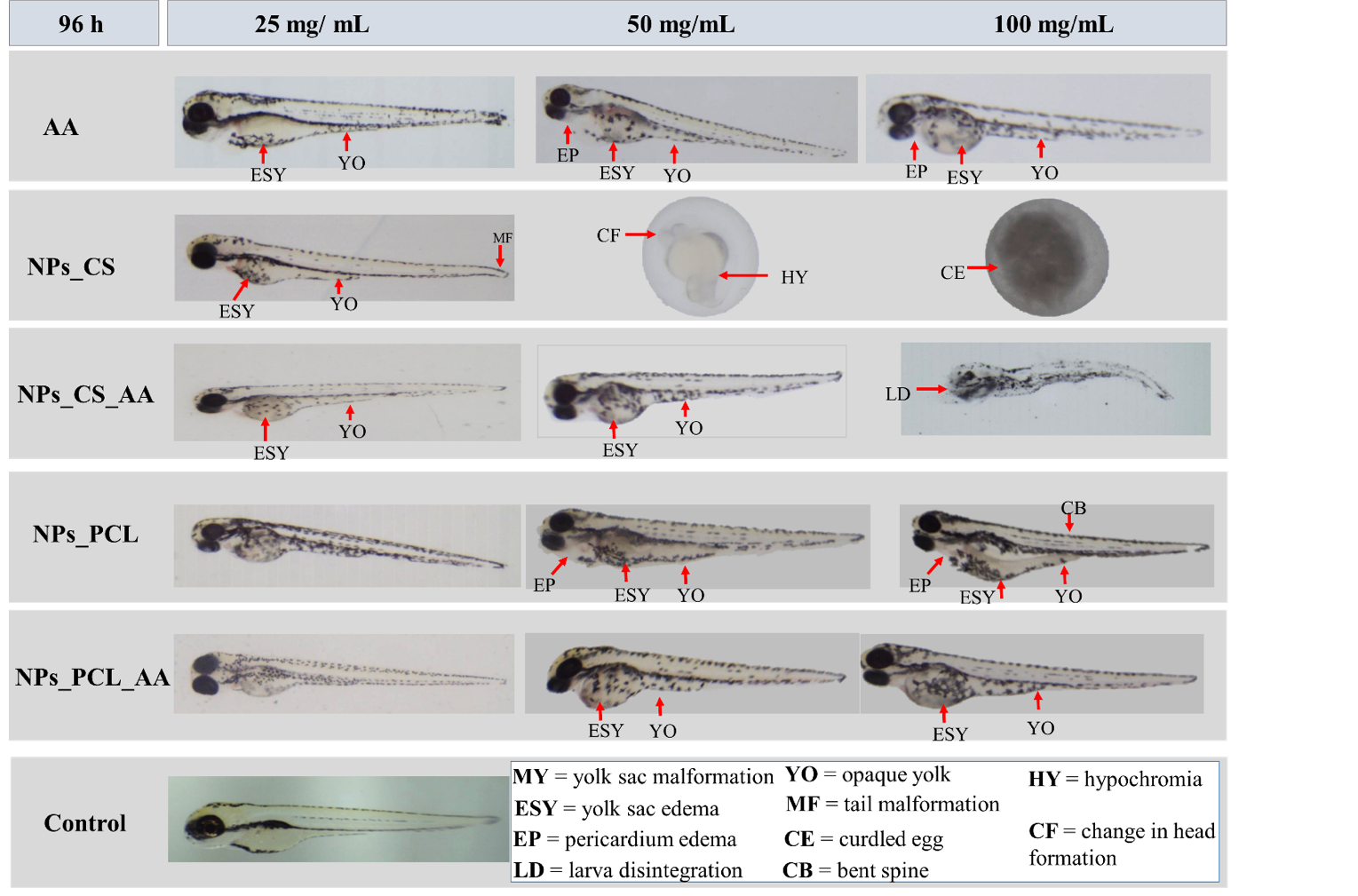


**Figure S2.** Morphological abnormalities (yolk sac malformation and edema, pericardial edema, opaque yolk, tail malformation, and bent spine) observed in the zebrafish embryos and larvae exposed to the different treatments: non-encapsulated ascorbic acid (AA), chitosan nanoparticles (NPs_CS), chitosan nanoparticles containing AA (NPs_CS_AA), PCL nanoparticles (NPs_PCL), and PCL nanoparticles containing AA (NPs_PCL_AA). The images were acquired using a stereomicroscope (Model SMZ 2 LED, Optika), at 2x magnification.


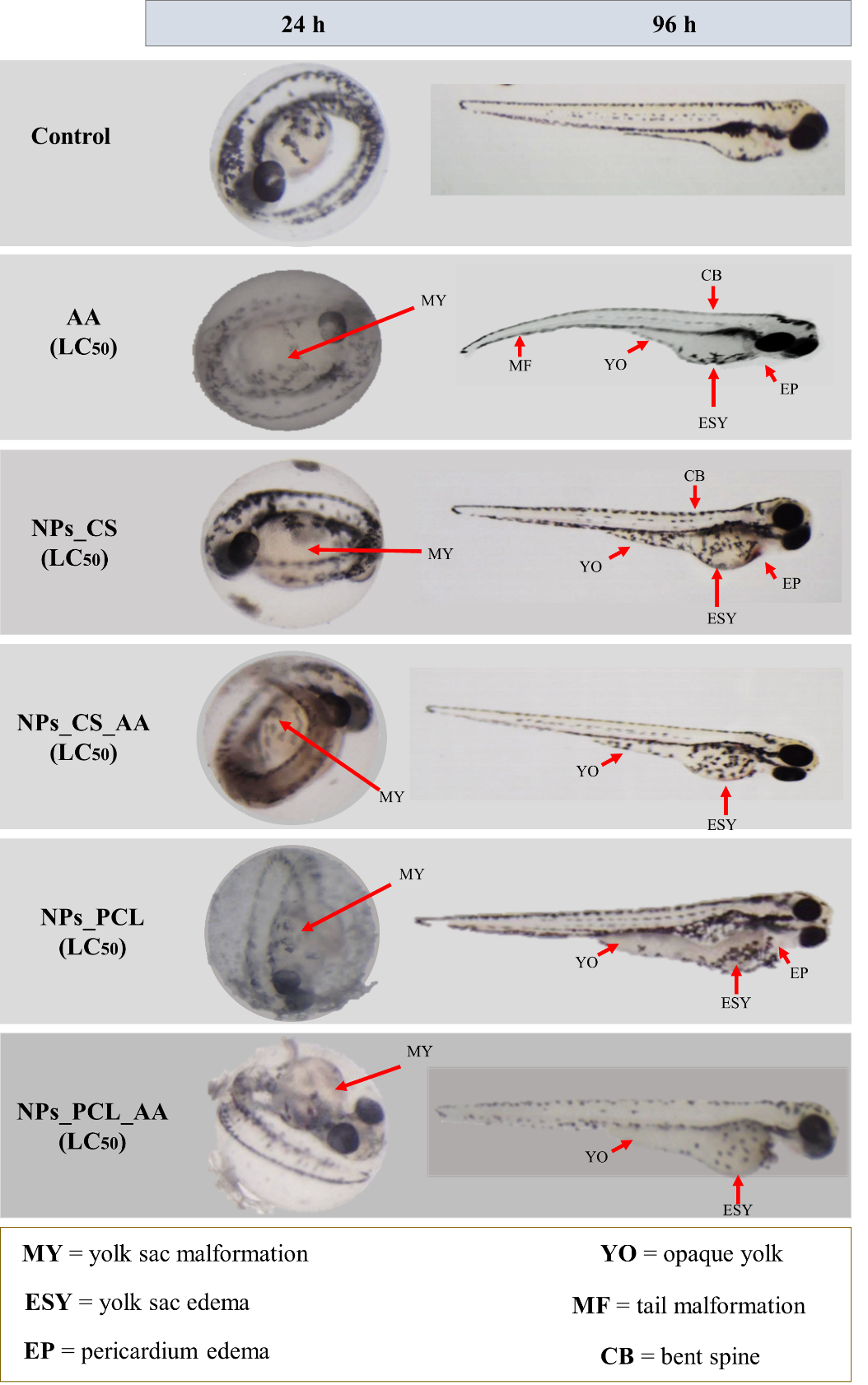

Supplement: Supplementary file 1 — Additional file 1: Figure S1. Morphological abnormalities (pericardial edema, skin lesions, undeveloped tail, tail alterations, and bent spine) observed in the zebrafish embryos and larvae exposed to the different treatments at concentrations of 25, 50, and 100 mg/mL: non-encapsulated ascorbic acid (AA), chitosan nanoparticles (NPs_CS), chitosan nanoparticles containing AA (NPs_CS_AA), PCL nanoparticles (NPs_PCL), and PCL nanoparticles containing AA (NPs_PCL_AA). The images were acquired using a stereomicroscope (Model SMZ 2 LED, Optika), at 2x magnification. Figure S2. Morphological abnormalities (yolk sac malformation and edema, pericardial edema, opaque yolk, tail malformation, and bent spine) observed in the zebrafish embryos and larvae exposed to the different treatments: non-encapsulated ascorbic acid (AA), chitosan nanoparticles (NPs_CS), chitosan nanoparticles containing AA (NPs_CS_AA), PCL nanoparticles (NPs_PCL), and PCL nanoparticles containing AA (NPs_PCL_AA). The images were acquired using a stereomicroscope (Model SMZ 2 LED, Optika), at 2x magnification. [file 12951_2021_910_MOESM1_ESM.docx]
